# Linking visceral fat accumulation to gut microbiota: key bacterial taxa and their roles in the glycogen synthesis pathway

**DOI:** 10.1038/s12276-026-01828-7

**Published:** 2026-09-10

**Authors:** Nam-Eun Kim, Beomki Cho, Yunjae Kim, Gihyeon Kim, Ga-Eun Yie, Haeon Kim, Jaesung Park, Hyun Kim, Chanyoung Jeong, Minji Lee, Yanghyun Joo, Sua Lim, Sihyeon Park, Yeji Hong, Kyungchan Min, Belong Cho, Jae Moon Yun, Eun Sung Jung, Yosep Ji, Seongbeom Cho, Je Kyung Seong, Hansoo Park, Jin-Ho Park, Sungho Won

**Affiliations:** 1 Institute of Health and Environment, Seoul National University, Seoul, Republic of Korea; 2 Department of Medical Informatics, Jeonbuk National University Medical School, Jeonju, Republic of Korea; 3 Department of Biomedical Science and Engineering, Gwangju Institute of Science and Technology, Gwangju, Republic of Korea; 4 Genomic Medicine Institute, Medical Research Center, Seoul National University, Seoul, Republic of Korea; 5 Department of Biomedical Sciences, Seoul National University College of Medicine, Seoul, Republic of Korea; 6 Interdisciplinary Program in Bioinformatics, Seoul National University, Seoul, Republic of Korea; 7 Department of Family Medicine, Seoul National University Hospital, Seoul, Republic of Korea; 8 Department of Family Medicine, Seoul National University College of Medicine, Seoul, Republic of Korea; 9 HEM Pharma, Gyeonggi-do, Republic of Korea; 10 College of Veterinary Medicine and Research Institute for Veterinary Science, Seoul National University, Seoul, Republic of Korea; 11 Laboratory of Developmental Biology and Genomics, Research Institute for Veterinary Science, and BK21 PLUS Program for Creative Veterinary Science Research, College of Veterinary Medicine, Seoul National University, Seoul, Republic of Korea; 12 Korea Model Animal Priority Center, Seoul National University, Seoul, Republic of Korea; 13 Genome and Company, Gyeonggi-do, Republic of Korea; 14 RexSoft Corp, Seoul, Republic of Korea; 15 Department of Public Health Sciences, Seoul National University, Seoul, Republic of Korea; 16 Present Address: Genome and Company, Gyeonggi-do, Republic of Korea

**Keywords:** Genetics research, Predictive markers, Metagenomics, Metagenomics

## Abstract

Obesity, marked by visceral fat accumulation, has a complex relationship with the gut microbiome that impacts body weight and fat accumulation. However, previous studies did not account for fat distribution, reflecting only overall fat mass, leaving specifics of this relationship partially understood. Here we analyzed the mechanistic links between visceral fat and the microbiome in a large cohort of healthy Koreans. Using permutational multivariate analysis of variance and prediction modeling, we examined associations between microbial profiles and metabolic variables including insulin, triglycerides, waist circumference and visceral fat. The strongest correlations were noted with specific enterotypes. Shotgun sequencing revealed that visceral fat is linked to the glycogen synthesis pathway influenced by *Dorea longicatena* and *Bifidobacterium adolescentis*. This suggests that these specific microbial signatures and their associated functional potential play a role in visceral fat-related obesity. To validate these findings, we conducted an in vivo study using diet-induced obesity mouse model. Oral administration of *D. longicatena* or *B. adolescentis* significantly promoted body weight gain and fat mass expansion and induced hepatic lipogenic gene upregulation. The prevalence of these strains in Korean and American populations highlights their global relevance, contributing to the development of personalized treatments and advanced health strategies.

## Gut microbiome links to visceral fat and obesity revealed

This study delves into the intricate relationship between gut microbiota and obesity, focusing on visceral fat, a key health risk factor. Researchers analyzed 16S rRNA sequencing data from 1,771 Koreans to explore microbiota’s impact on 52 clinical variables, identifying obesity-related markers. They defined an obesity index using principal component analysis and confirmed associations with metagenomic sequencing. Key findings include the identification of *Dorea longicatena* and *Bifidobacterium adolescentis* as significant contributors to obesity, with *D. longicatena* linked to glycogen synthesis pathways. These bacteria, when administered to mice, led to increased body weight and visceral fat, highlighting their role in obesity. This comprehensive analysis suggests potential microbial targets for obesity management, emphasizing the need for further research to explore therapeutic applications and validate these findings across diverse populations.

This summary was initially drafted using artificial intelligence, then revised and fact-checked by the author.

## Introduction

The relationship between gut microbiota composition and obesity has been extensively explored^1,2^. A higher body mass index (BMI) is associated with decreased diversity in gut microbiota and a lower proportion of Bacteroidetes compared with these associations with Firmicutes^1,3–5^. Different types of adipose tissue interact bidirectionally with distinct microbiota^6^. Such changes in the gut microbiota of obese individuals increase the production of short-chain fatty acids, which enhance energy harvest and reverse cholesterol transport via bile acid circulation^7,8^.

Obesity can be measured using various indices beyond BMI, such as visceral fat, which is closely linked to a high risk of health issues, including inflammation, cardiovascular disease and fatty liver. Among obesity-related measures, including subcutaneous fat, waist circumference (WC), waist-to-hip ratio, hipline and BMI, visceral fat has the strongest association with microbial species^9–12^. Studies using TwinsUK data have observed that gut bacteria promote visceral fat accumulation by producing short-chain fatty acids^13,14^, and Mendelian randomization analyses using genetic data have revealed a causal relationship between Lachnosiraceae and visceral fat^15^. Regarding East Asian cohorts, correlations have been observed between 16S rRNA data and visceral fat in Japanese individuals, as well as strong associations between *Bifidobacterium* and *Blautia* and visceral fat in 372 Chinese individuals^16,17^. Although previous studies have investigated the link between visceral fat and the microbiome, to our knowledge, no comprehensive analysis using a large cohort of healthy individuals has been performed. Obesity is a multifactorial condition influenced by genetic and environmental factors, and addressing it requires comprehensive approaches that consider these various influences.

Multiple measures, such as BMI, WC and waist-to-hip ratios, provide different insights into a person’s body composition. BMI and WC are easily measured and have been the standard for defining obesity. BMI is a universally applicable and accessible measure for adults of all ages and sexes, making it a valuable indicator at the population level. The World Health Organization also defines ‘overweight’ and ‘obese’ on the basis of BMI^18^. However, despite their practicality, these measures do not account for fat distribution, reflecting only overall fat mass. Conversely, visceral fat is a more important contributor to a range of health conditions, including cardiovascular and cerebrovascular diseases, hypertension, type 2 diabetes and all-cause mortality^19–24^. Visceral adipose tissue has a higher number of beta-adrenergic receptors than subcutaneous adipose tissue^25^, leading to more efficient fat breakdown and increased secretion of free fatty acids^26^, which promote insulin resistance and related health complications^27,28^. Each of these measures has limitations and may not provide a complete picture of an individual’s health risk. Therefore, it is beneficial to consider multiple measurements when investigating the effect of gut microbiota on obesity.

In this study, we focused on two primary goals. First, we examined the relationship between gut microbiota and 52 clinical variables using 16S rRNA sequencing data from 1771 Koreans. We also defined a standard list of microbiota strains in Koreans and compared them with a standard list of microbiota strains identified in North Americans to determine the species common across populations and those specific to Koreans^29^. Second, we selected obesity-related variables from the 52 clinical variables and defined an obesity index using principal component (PC) analyses. We then investigated the effect of gut microbiota on obesity and identified candidate metagenomic markers. The association of these markers was further confirmed with metagenome shotgun sequencing data and elucidating their underlying functional mechanisms and pathways related to obesity and visceral fat accumulation. Currently, obesity treatment is centered around surgery, incretin-based therapies^30^, or medications such as semaglutide or liraglutide^31^. This study represents one of the most comprehensive investigations so far on the impact of microbiota on multiple clinical variables in East Asians, presenting new insights for obesity management and personalized treatments.

## Materials and methods

### Participants

This study included 1833 donors who participated in the health screening program at Seoul National University Hospital from February 2017 to November 2018. These donors underwent various medical evaluations, including physical examinations, blood tests, abdominal computed tomography scans, abdominal ultrasonography, questionnaires and stool sample collections. All 1833 eligible donors were confirmed to be free of any serious illnesses at the time of examination, and their stool samples were analyzed to ascertain the composition of their microbiome. We excluded 62 participants owing to missing values for 45 or more clinical variables, leaving 1771 participants whose stool samples were subjected to 16S rRNA analysis. This study was approved by the Institutional Review Board of Seoul National University Hospital (IRB No. C-1609-051-790) and conducted in accordance with the Declaration of Helsinki. Informed consent was obtained from all participants before they took part in the research.

### 16S rRNA sequencing and bacterial profiling

All fecal samples were stored at −80 °C until DNA extraction, which was performed using the FastDNA SPIN Kit for Soil (MP Biomedicals) according to the manufacturer’s protocol. The purity and concentration of the extracted DNA were assessed using a NanoDrop One Spectrophotometer (Thermo Scientific). The V3–V4 hypervariable regions of the 16S rDNA gene were amplified using specific primers: 16S_V3_F (5′-TCGTCGGCAGCGTCAGATGTGTATAAGAGACAGCCTACGGGNGGCWGCAG-3′) and 16S_V4_R (5′-GTCTCGTGGGCTCGGAGATGTGTATAAGAGACAGGACTACHVGGGTATCTAATCC-3′). The polymerase chain reaction (PCR) products were used to prepare libraries according to the MiSeq System guide (Illumina) and quantified with QIAxpert (QIAGEN). The amplicons were then pooled at equimolar ratios and sequenced on a MiSeq platform (Illumina).

Paired-end reads that matched the adapter sequences were trimmed using Cutadapt version 1.17 with a minimum overlap of 11 bp, a maximum error rate of 15%, and a minimum length of 10 bp (ref. ^32^). The resulting FASTQ files containing paired-end reads were merged with CASPER version 0.8.2 with a mismatch ratio of 0.27 and then subjected to quality filtering with the Phred (Q) score-based criteria described by Bokulich^33^. Any reads shorter than 350 bp and longer than 550 bp after merging were discarded. To identify chimeric sequences, VSEARCH version 2.3.0 was used to perform a reference-based chimera detection step against the SILVA gold database^34,35^. Sequence reads were clustered into operational taxonomic units (OTUs) using VSEARCH with an open clustering algorithm under a threshold of 97% sequence similarity. The representative sequences of the OTUs were finally classified using the EzBioCloud database with UCLUST (parallel_assign_taxonomy_uclust.py script on QIIME version 1.9.1) under default parameters^36^.

### Enterotyping of participants

The relative abundance of each taxon was log-transformed, and participants were clustered into four statistically optimal groups based on their gut microbiome composition at the genus level using the Dirichlet multinomial mixture model^37^. We evaluated the impact of clinical variables substantially explained by the enterotypes through linear regression and *F*-tests. The Kruskal–Wallis test was used to determine enterotypes that exhibited differential levels of each log-transformed clinical variable and bacterial taxon at both genus and species levels.

### Defining the obesity score

Hierarchical clustering analysis was performed on 52 clinical variables using the hclust() function in R. The resulting clusters were annotated with selected clinical variables. We identified the cluster containing obesity-related variables and carried out PC analysis on these variables. The selected cluster included nine clinical variables, and the first PC (PC1), which explained the largest proportion (50.4%) of variance, was chosen as the obesity-related score. This score is referred to as the obesity score throughout this Article. It is important to note that this score represents a composite metabolic phenotype of obesity based on multiple clinical variables, rather than the standard clinical BMI threshold of 30.

### Shotgun metagenome sequencing

To complement the findings from 16S rRNA data, we performed a shotgun metagenomics analysis focusing on the association with obesity. We selected 100 participants with the highest obesity scores to represent the high-obesity-score group and matched them with 100 participants with the lowest scores based on age and sex. The matching process ensured no significant differences in age and sex between the groups. Upon assessing data quality, we found that 23 participants from the high-score and 25 from the low-score groups met our quality standards, resulting in metagenomics sequences for 48 participants using the Nextera DNA Flex Library Prep Kit and Illumina Novaseq. Strict quality control measures—including a stool sample quantity larger than 0.3 µg, sample purity ratio greater than 1.6 (absorbance at 260 nm divided by the absorbance at 280 nm), DNA integrity number above 6, and low correlations (below 0.3) between the 16S rRNA and shotgun data—were applied, leading to 44 participants, 22 from the high-score and 22 from the low-score group, being deemed suitable for further analysis.

For quality control of paired-end reads, KneadData was used for initial processing and Trimmomatic for adapter trimming. Reads were aligned to GRCh38 using Bowtie2 to eliminate human genome contamination. Next, HUMAnN 3.0 and MetaPhlAn3 were used to profile microbial metabolic pathways and community composition, respectively, using UniRef90 and ChocoPhlAn DB for the analysis. All analyses were conducted with the default settings of each program.

### Bacteria culture

*Dorea longicatena* (DSMZ 13814) was purchased from the Leibniz Institute DSMZ-German Collection of Microorganisms and Cell Cultures, and *Bifidobacterium* *adolescentis* (KCTC 3216) was obtained from the Korean Collection for Type Cultures. *D. longicatena* was grown in tryptic soy broth (BD Difco) supplemented with 0.05% L-cysteine (Sigma-Aldrich), whereas *B. adolescentis* was grown in BL broth (MBcell) supplemented with 0.05% L-cysteine. All bacterial species were cultured at 37 °C under anaerobic conditions. Colony-forming units (CFU) in each culture were counted, and the concentrate was supplemented with 20% glycerol stored at −80 °C before use.

### Mouse experiment

The mouse experiment was conducted according to the approved protocols of the Institutional Animal Care and Use Committee of GIST (GIST-2023-060). Six-week-old male C57BL/6 mice were purchased from G-bio. Before bacterial treatment, mice were orally administered 200 μl of antibiotics solution (ampicillin 10 mg/ml, neomycin 10 mg/ml, vancomycin 5 mg/ml) daily for one week. Following a 1-week washout period, mice were fed high-fat diet (Research diets, D12492) during the remaining experimental period. Mice were orally treated with 10^8^ CFU of *D. longicatena* or *B. adolescentis* with phosphate-buffered saline (PBS), whereas control mice were treated with PBS every day. Body weight and food intake were measured every week. Prior to sacrifice, fecal samples were collected and frozen at −80°C, and body composition was analyzed using dual energy X-ray absorptiometry (Medikors, InAlyzer). Upon euthanasia, blood, liver, inguinal white adipose tissue (iWAT) and epididymal white adipose tissue (eWAT) were collected. For subsequent RNA isolation, the tissue samples were stored in RNAlater and maintained at −20 °C. Blood glucose was measured with glucometer (Accu-Chek). Serum glucose (Abcam, ab65333), triglyceride (TG; Abcam, ab65336), cholesterol (Abcam, ab65390) and free fatty acid (Abcam, ab65341) were measured with assay kits, according to manufacturer’s protocols. To compare the mean values of metabolic parameters among mice treated with *D. longicatena*, *B. adolescentis* or PBS as a vehicle control, we performed one-way analysis of variance.

### Linear discriminant analysis effect size (LEfSe)

To further investigate the association between microbial taxa and obesity-related traits, we conducted LEfSe using the Galaxy platform. This analysis identified microbial taxa enriched in high-risk versus low-risk group based on obesity-related parameters, including WC, BMI, total cholesterol, high-density lipoprotein (HDL) cholesterol, low-density lipoprotein (LDL) cholesterol and TG.

### RNA isolation

Tissue samples were homogenized with Lysing matrix E (MP Biomedicals). Tissue homogenates were mixed with TRIzol reagents (Thermo Scientific) and RNA was isolated according to manufacturer’s instructions. One microgram RNA was used to synthesize cDNA for subsequent quantitative PCR.

### Statistical analyses

#### Goodness of fit and prediction modeling

Permutational multivariate analysis of variance (PERMANOVA) was used to calculate the goodness of fit (*R*²) of the metagenome in relation to clinical variables using the R package vegan^38^. In addition to age, sex, region and batch information, the goodness of fit of clinical variables was further assessed in the context of beta diversity (Bray–Curtis distance) calculations and ranked accordingly. Next, we focused on prediction modeling. Clinical variables were transformed into binary variables using their median values and predicted using covariates and microbial species. We considered 21 supervised learning models, including Adaboost, elastic net, gradient boosting, linear support vector machine (SVM), auto SVM, orthogonal matching pursuit, linear discriminant analysis, quadratic discriminant analysis, naive Bayes, Bernoulli naive Bayes, Gaussian Ridge, Lasso, Lars, LassoLars, extra tree classifier, decision tree classifier, logistic regression, random forest, XGBoost, stochastic gradient descent (SGD) and multi-layer perceptron (MLP) from the scikit-learn package (Python 3). The best model was selected based on the highest mean under the curve (AUC) after 10-fold cross-validation^39^, for predicting each phenotype with taxa and covariates. We compared the mean AUC of each variable when predicted using only covariates (Covariates only) versus when predicted using both covariates and microbial species (Covariates + Gut microbiota), using Delong’s test for prediction in addition to covariates.

#### Association analysis of 52 clinical variables with 16S rRNA sequencing data

Species associated with 52 clinical variables were identified using the Phylogenetic tree-based microbiome association test (TMAT)^40^, adjusting for age, sex, region and batch effects. For association analyses, only OTUs present in more than 50% of participants and representing >0.5% of the total read count were considered. The results of statistical tests were deemed significant based on multiple-testing-adjusted *P* values using the Benjamini–Hochberg method (BH *P*).

#### Shotgun metagenome sequencing analyses

Community-encoded functions were quantified with HUMAnN 3.0, and the network of microbial species was drawn with OmicsNet^41^. Alpha and beta diversities were compared between high and low visceral fat groups. Alpha diversity was assessed using observed Chao1 and Shannon indices, whereas beta diversity was determined using a principal coordinates analysis plot with Jaccard distance. Pathways determined using HUMAnN 3.0 utilizing the MetaCyc database were filtered by the following criteria^42^: present in over 75% of individuals and comprising less than 25% of unclassified species within those individuals. Contributional diversities of filtered pathways were calculated as reported earlier^42^. Within-sample contributional diversity increases as more species contribute to a function within a single sample, whereas between-sample contributional diversity increases as the types of species contributing to the function differ across samples. Linear regression analyses were performed to investigate whether the filtered pathways showed a significant association with candidate taxa and obesity scores. Finally, for the significant pathways, the Kyoto Encyclopedia of Genes and Genomes (KEGG) pathway map and module were examined to elucidate the underlying mechanisms.

## Results

### Descriptive statistics

Baseline characteristics of the 55 clinical variables are presented in Supplementary Table 1. The average age of the participants was 58.05 years (standard deviation (s.d.) 11.43), with women accounting for 45.2% of the cohort. The age range spanned from 20 to 84 years. The mean BMI was 23.8 kg/m^2^ (s.d. 3.09), with values ranging from 14.4 to 39.5 kg/m^2^. Percent body fat averaged 26.08% (s.d. 6.28). WC had a mean of 85.42 cm (s.d. 9.25) and the mean visceral fat area was 126.64 cm² (s.d. 49.32).

### Assessing clinical variables to explain and predict the gut microbiome

**Fig. 1:**
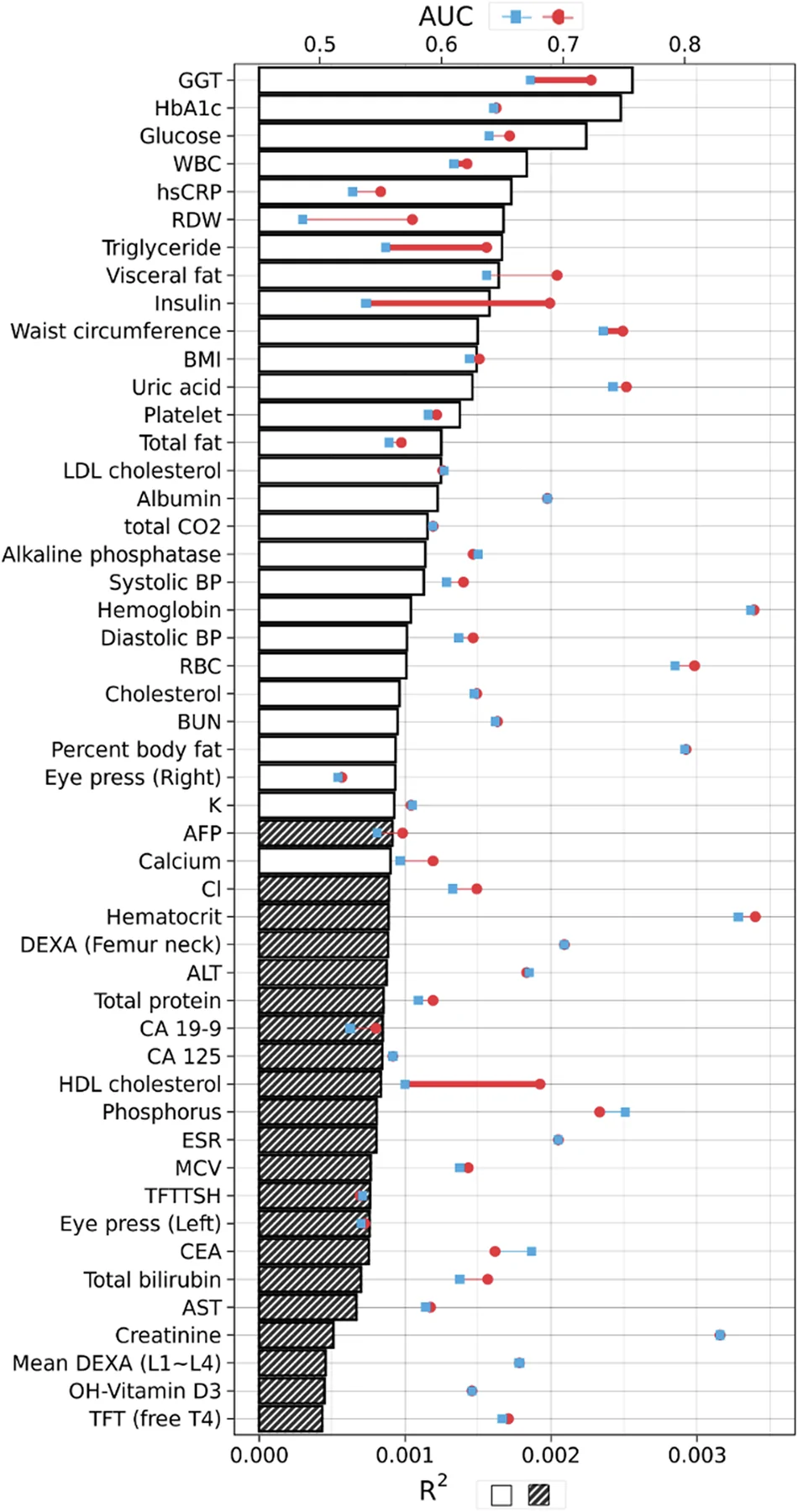
Goodness of fit (*R*^2^) and AUC values for clinical variables using 16S rRNA data. The bars display the goodness of fit (*R*²) of the clinical variables. Empty and hatched bars signify the Bonferroni-adjusted *P* values that are less and larger than 0.05, respectively. Solid blue rectangles and red circles represent the AUCs of models ‘Covariates only’ and ‘Covariates + Gut microbiota’, respectively. The red line connecting two dots represents the *P* of DeLong’s test for comparing AUCs, whereas bold lines represent Bonferroni-adjusted *P* of less than 0.05.

Figure 1 illustrates the prediction accuracy for various clinical variables. It is important to note that the *R*² values for the clinical associations were relatively small (all <0.01), yet remained statistically significant (BH *P* < 0.05). Gamma-glutamyl peptidase (GGT) exhibited the highest *R*² and AUC values. Adding GGT as a covariate alongside age and sex in the PERMANOVA model increased the *R*² for the gut microbiota by 0.0026 (BH *P* = 0.004). When comparing the AUC of GGT predicted using ‘Covariates only’ versus ‘Covariates + Gut microbiota’, the 10-fold cross-validation AUC significantly increased from 0.673 to 0.723 (Delong’s test *P* < 0.001). Variables such as visceral fat, insulin, WC, uric acid, white blood cell count (WBC), TG and systolic blood pressure (SBP) also showed high *R*² or AUC values. Notably, insulin and TG exhibited the largest AUC increases when 16S rRNA microbiome data were incorporated, improving from 0.538 to 0.689 and from 0.554 to 0.637, respectively. Significant AUC increases were observed for hematocrit, red blood cell count, uric acid, WC, phosphorus, GGT, visceral fat, insulin, HDL cholesterol, glucose, TG, cholesterol, chlorine (Cl) and carcinoembryonic antigen when gut microbiota data were included. HDL cholesterol showed no significant *R*² for microbial species but had a significantly increased AUC due to microbial species, whereas BMI showed no significant increase in AUC and had a significant *R*².

### Enterotypes of participants and their characteristics

**Fig. 2:**
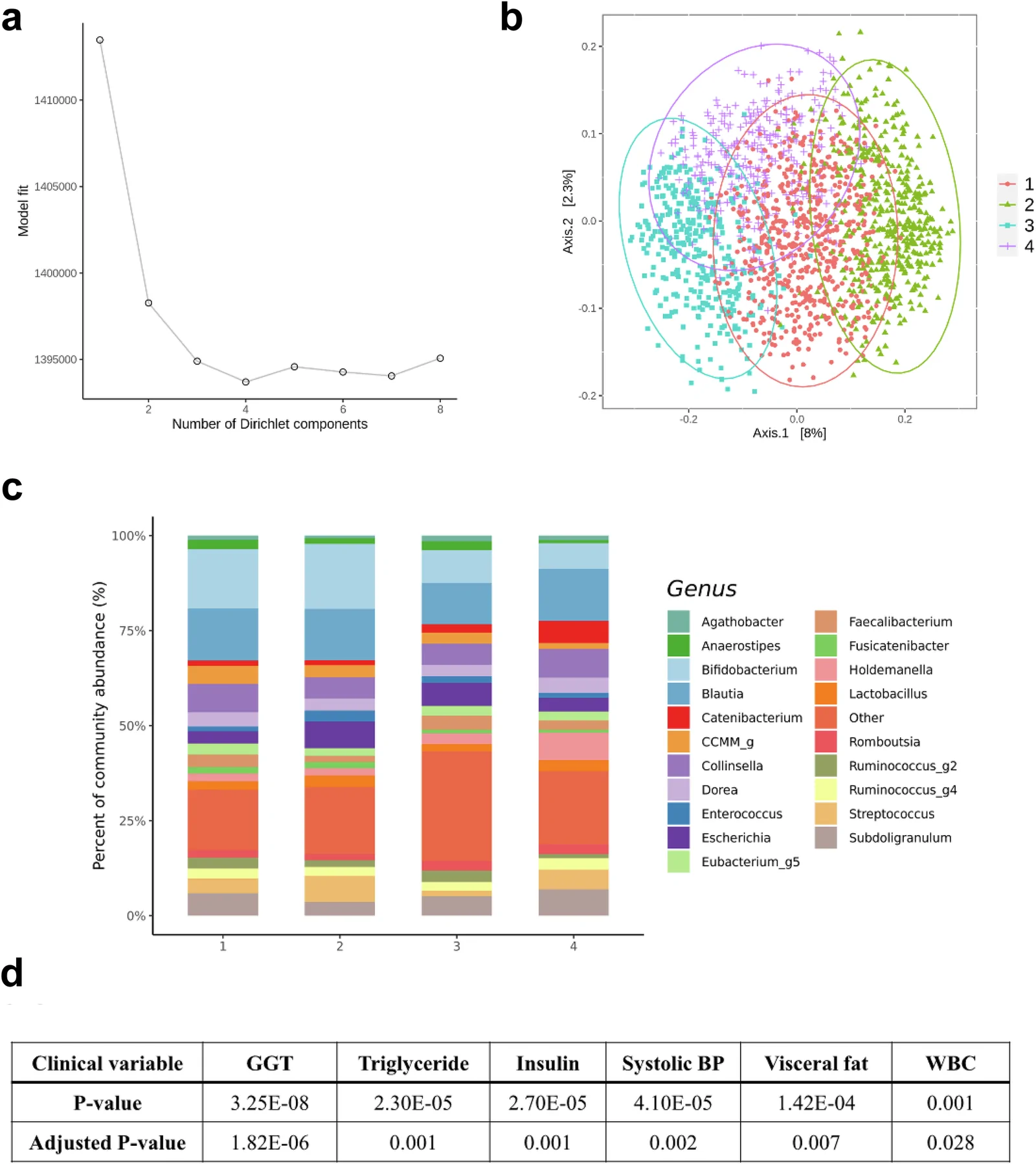
16S rRNA enterotypes of the participants. **a**, Goodness of fit by the number of Dirichlet multinomial mixtures using Laplace criteria. **b**, PCoA plot using UniFrac distance by enterotype. **c**, Plot showing relative abundance at the genus level. **d**, Clinical variables were compared among four enterotypes with linear regression and an *F*-test.

Participants were clustered into groups based on 16S rRNA sequencing data, revealing four statistically optimal clusters based on the Dirichlet multinomial mixture model (Fig. 2a). While visual overlap exists in the principal coordinate analysis (PCoA) plot due to the large cohort size, PERMANOVA confirmed significant differences in community structure among these groups (*P* < 0.001, Fig. 2b). Notably, *Bifidobacterium* genus proportions were similar within each pair but significantly different between the pairs. Enterotype 2 exhibited a higher prevalence of *Enterococcus, Escherichia* and *Streptococcus* compared with enterotype 1. Enterotype 3 had a notable presence of unclassified genera, whereas enterotype 4 was abundant in *Catenibacterium* and *Holdemanella* (Fig. 2c).

Comparisons of clinical variables among the four enterotypes using linear regression and *F*-tests identified SBP, visceral fat, GGT, TG, insulin and WBC as having the most significant associations (Fig. 2d and Supplementary Table 2). These variables also demonstrated high *R*² and AUC values (Fig. 1). A comprehensive mean difference test on clinical variables revealed significant differences across enterotypes (Supplementary Table 3). Notably, enterotype 3 exhibited the lowest levels of metabolic risk factors, including visceral fat and insulin, highlighting the clinical relevance of the identified enterotypes. Conversely, enterotype 4 displayed the highest values, indicating a greater risk. Significant differences between enterotype 3 and 4 were observed for the genera *Anaerostipes*, *Dorea* and *Escherichia*. At the species level, *Dorea longicatena* exhibited notable variance between enterotype 3 (median 0.016) and enterotype 4 (median 0.023; Supplementary Table 5).

### Association analysis

**Fig. 3:**
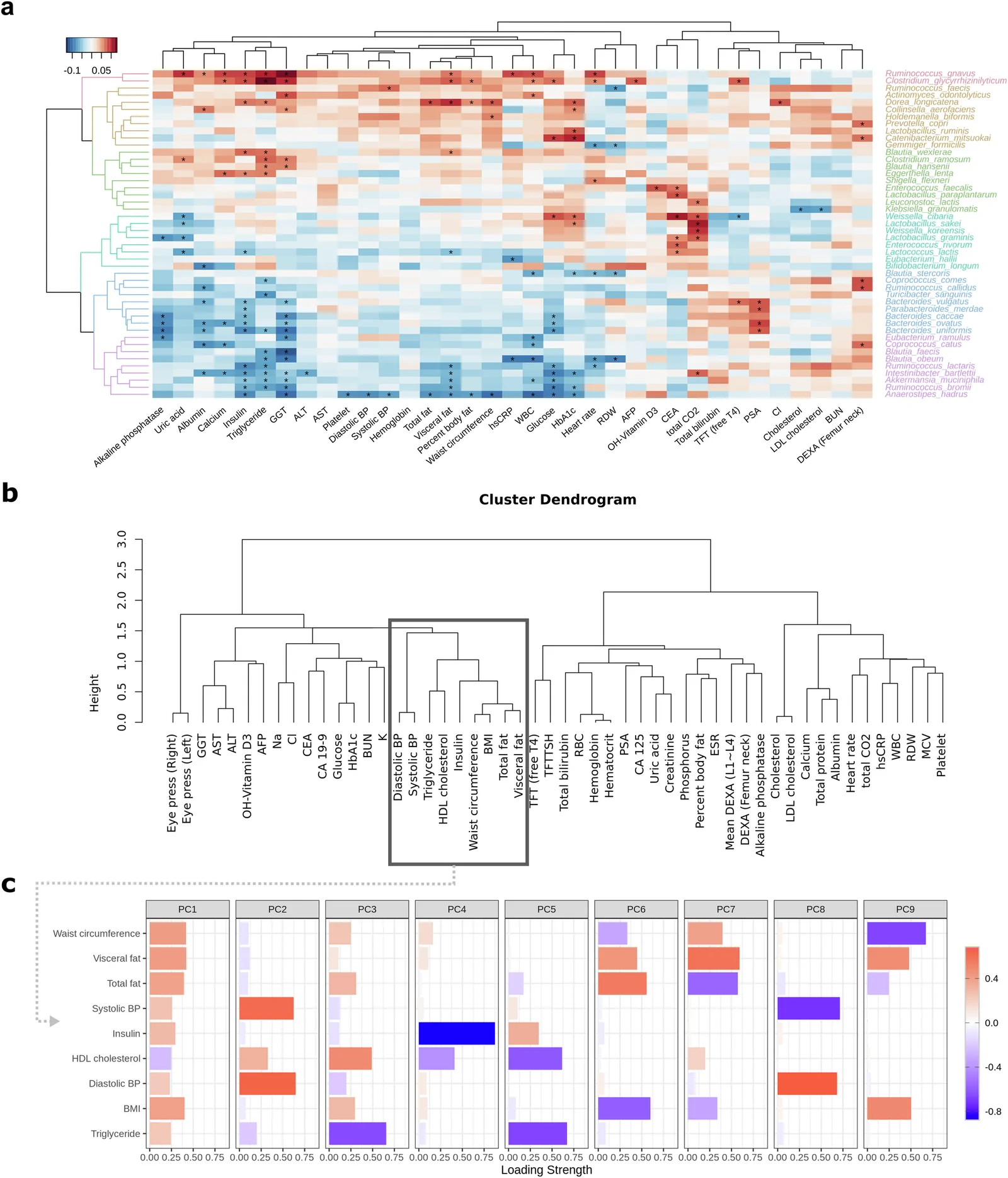
Heatmap of microbial association and hierarchical clustering with PC analysis (PCA) loading factors. **a**, The color of each cell represents the effect size based on the TMAT analysis result, with red and blue indicating positive and negative directions, respectively. An asterisk is added if the adjusted *P* is less than 0.05. A hierarchical clustering analysis was conducted among species, and those exhibiting similar relationships with clinical variables are represented by the same text color. **b**, Dendrogram from hierarchical clustering of clinical variables. **c**, PCA loading factors of nine PCs were classified into the same cluster across clinical variables. The length and color of the bar represent the strength and direction of the loading factor, with red and blue indicating positive and negative loading, respectively.

Three distinct statistical methods were used to assess microbiome associations after adjusting for covariates. Eight species displayed significant associations with visceral fat, TG, insulin and obesity scores (Fig. 3a). *Ruminococcus gnavus*, *Clostridium glycyrrhizinilyticum*, *Dorea longicatena* and *Blautia wexlerae* demonstrated positive associations, whereas *Blautia obeum*, *Ruminococcus lactaris*, *Intestinibacter bartlettii*, *Akkermansia muciniphila* and *Ruminococcus bromii* showed negative associations. Notably, *D. longicatena* and *Anaerostipes hadrus* exhibited positive and negative associations, respectively, with total fat, visceral fat, percent body fat, WC and HbA1c. Among the variables investigated, visceral fat was significantly associated with the largest number of taxa, whereas BMI showed no significant association with any taxa after the Benjamini–Hochberg multiple adjustments.

Hierarchical clustering revealed that both clinical variables and microbial taxa were categorized based on similarity, with taxa within the same cluster identified by a consistent color. For example, the pink cluster, which included *Ruminococcus gnavus*, and the yellow cluster, containing *Ruminococcus faecis*, displayed similar patterns. By contrast, the purple *Eubacterium ramulus* presented a distinctly different pattern.

### Species associated with obesity score

Hierarchical clustering was applied to 52 clinical variables. The cluster dendrogram in Fig. 3b demonstrates that SBP, diastolic blood pressure (DBP), TG, HDL cholesterol, insulin, WC, BMI, total fat and visceral fat were highly correlated, forming a common cluster. Because all these variables are associated with obesity, we performed PC analysis using them; the cumulative variance exceeded 85% up to the fourth PC.

Loading factors for the obesity score (PC1) were positive and highly similar among all metabolic variables except for HDL cholesterol, which was negatively correlated, reflecting its inverse relationship with obesity (Fig. 3c). PC2 exhibited a strong correlation with SBP and DBP, whereas PC4 reflected the inverse effect of insulin. Intriguingly, PC7 displayed a strong positive correlation with visceral fat and WC and a significant negative correlation with total fat and BMI. This could indicate ‘skinny fat’—a body composition that, despite appearing healthy, carries a high risk of metabolic diseases due to high fat content. The variance of PC7 to PC9 was quite small, and no significant result was observed.

Using PC scores as outcomes, we identified candidate species significantly associated with PC scores using TMAT (Supplementary Table 4). PC1, defined as an obesity score, was positively correlated with *Ruminococcus gnavus* (*β*_TMAT_ = 0.029, BH *P* = 0.019), *D. longicatena* (*β*_TMAT_ = 0.045, BH *P* < 0.001) and *Clostridium glycyrrhizinilyticum* (*β*_TMAT_ = 0.038, BH *P* = 0.002), but negatively correlated with *Anaerostipes hadrus* (*β*_TMAT_ = −0.054, BH *P* < 0.001), *Ruminococcus lactaris* (*β*_TMAT_ = -0.035, BH *P* = 0.008), *Bacteroides uniformis* (*β*_TMAT_ = −0.036, BH *P* = 0.007) and several other taxa. PC4, representing negative insulin, had a significant negative association with *Ruminococcus gnavus* (*β*_TMAT_ = −0.089, BH *P* = 0.024), reflecting its positive association with obesity. *Eggerthella lenta* (*β*_TMAT_ = −0.106, BH *P* = 0.007) had a similar effect on PC4, reflecting negative insulin despite no significant association with PC1. Following the identification of obesity-associated taxa via 16S rRNA screening (Supplementary Table 4), we performed shotgun metagenomic sequencing to investigate the functional pathways of these candidates.

### Metagenomic distribution among the Korean population revealed through shotgun sequencing

Because obesity-related variables showed strong correlations with gut microbiota in healthy populations, as determined on the basis of 16S rRNA gene sequencing data, we further analyzed the metagenomic taxa of 44 participants selected on the basis of their obesity score.

Comparing the distribution of 16S rRNA gene sequencing and shotgun metagenomics sequencing data, we observed a decrease in the dominant phylum Firmicutes and an increase in Actinobacteria in shotgun metagenomic sequencing data. This difference may be attributed to the average 16S rRNA copy number in Firmicutes being 6.9 as opposed to 3.2 in Actinobacteria, which may have resulted in an approximately 2-fold difference (Supplementary Fig. 1a). Similarly, the prevalence of genus *Blautia* decreased, whereas that of *Bifidobacterium* increased, potentially correlating with their average 16S rRNA copy numbers of 5.4 and 3.2, respectively (Supplementary Fig. 1b). The profiling databases used in the two datasets also varied (EzBioCloud versus ChocoPhlAn).

**Fig. 4:**
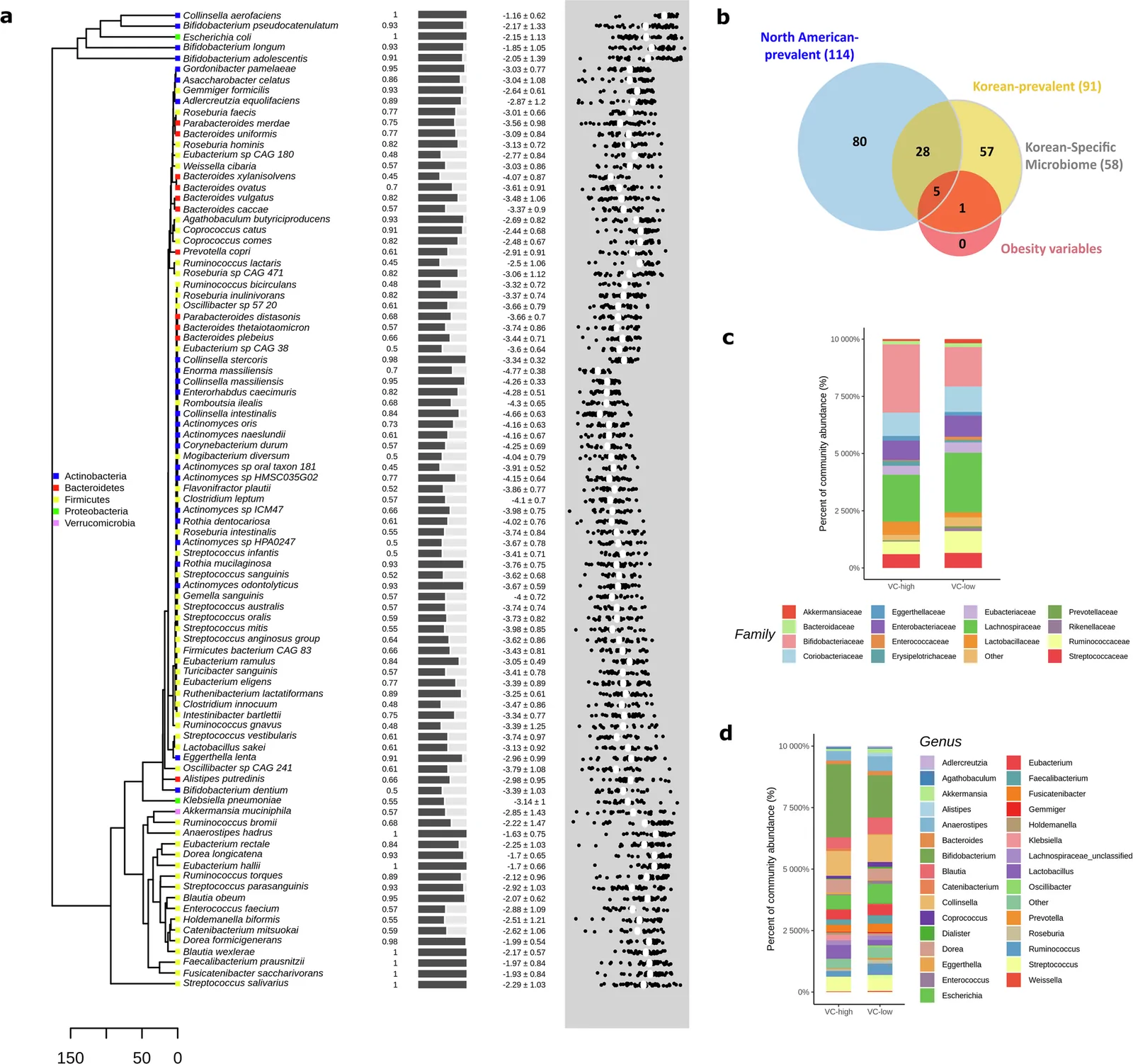
Shotgun sequencing revealed the complex gut bacterial community. **a**, A hierarchical tree of the 91 most prevalent species was identified on the basis of the shotgun sequencing data from 44 participants. The phylum of each species is denoted by colored squares: Actinobacteria (blue), Bacteroidetes (red), Firmicutes (yellow), Proteobacteria (green) and Verrucomicrobia (purple). The prevalence, proportion of individuals in whom the species is present, and relative abundances of each species are also shown. For each species, a white line indicates the mean of the distribution of log10-transformed relative abundance, shown as a jitter plot. **b**, A Venn diagram representing the list of standard strains present in North American healthy individuals as reported by Cheng et al.^29^ (blue circle), standard species lists for Korean healthy individuals (yellow circle) and species related to the obesity score (PC1; red circle). **c**,**d**, Stacked bar plots of relative abundance of family and genus for two groups based on visceral fat: VC high (participants with a larger visceral fat value than the median) and VC low (participants with a smaller visceral fat value than the median).

In the metagenome sequencing data, approximately 20% (91/473) of species were present in over 45% of participants, indicating their prevalence and abundance in the gut of the Korean population. This result aligns with the standard gut microbial community of healthy North American individuals^29^. The hierarchical tree and distribution of the Korean microbiota are depicted in Fig. 4a and their network with metabolites is shown in Supplementary Fig. 2. Within Firmicutes, species marked with a yellow dot were observed most frequently (Fig. 4a). Species within the same phylum are generally clustered together in the hierarchical clustering analysis. *Dorea longicatena*, with a high prevalence of 0.93 and a relatively high value for the log of relative abundance, is frequently noted in the Korean population. Conversely, some taxa, such as *Collinsella intestinalis*, exhibited a prevalence of 0.84 but showed very low abundance. As shown in Supplementary Fig. 2, the network visualizes the functional metabolic interactions of these prevalent species. While the analysis began with the 91 species present in over 45% of Korean participants, the resulting network specifically highlights 57 species with predicted metabolic links to host metabolites. The first cluster centers around *Collinsella aerofaciens*, *Anaerostipes hadrus* and *Streptococcus salivarius* species, whereas the second cluster centers around *Blautia wexlerae*, *Faecalibacterium prausnitzii* and *Bacteroides ovatus*, among others.

### Most common metagenomic taxa in healthy Korean population

We identified 91 bacterial species as the standard gut microbiota in healthy Koreans and compared them with the North American reference list (Fig. 4a). Among the 91 candidate species of the Korean cohort, 33 species were shared with the North American standard gut microbial community (Fig. 4b). Five of these shared species, *Alistipes putredinis*, *Eubacterium eligens*, *Intestinibacter bartlettii*, *Catenibacterium mitsuokai* and *Dorea longicatena*, were significantly associated with PC1 and visceral fat. In addition, we identified 58 species specific to the Korean microbiome, among which *Bifidobacterium adolescentis* was significantly associated with PC1 and visceral fat (Supplementary Table 8).

We analyzed the distribution of gut microbiota at the family and genus levels in 44 healthy Koreans (Fig. 4c, d). Several dominant species showed a significantly higher abundance in the group with higher visceral fat (VC high) than in the group with lower visceral fat (VC low).

### Metagenomic and contributional diversity of the taxa and related pathways

**Fig. 5:**
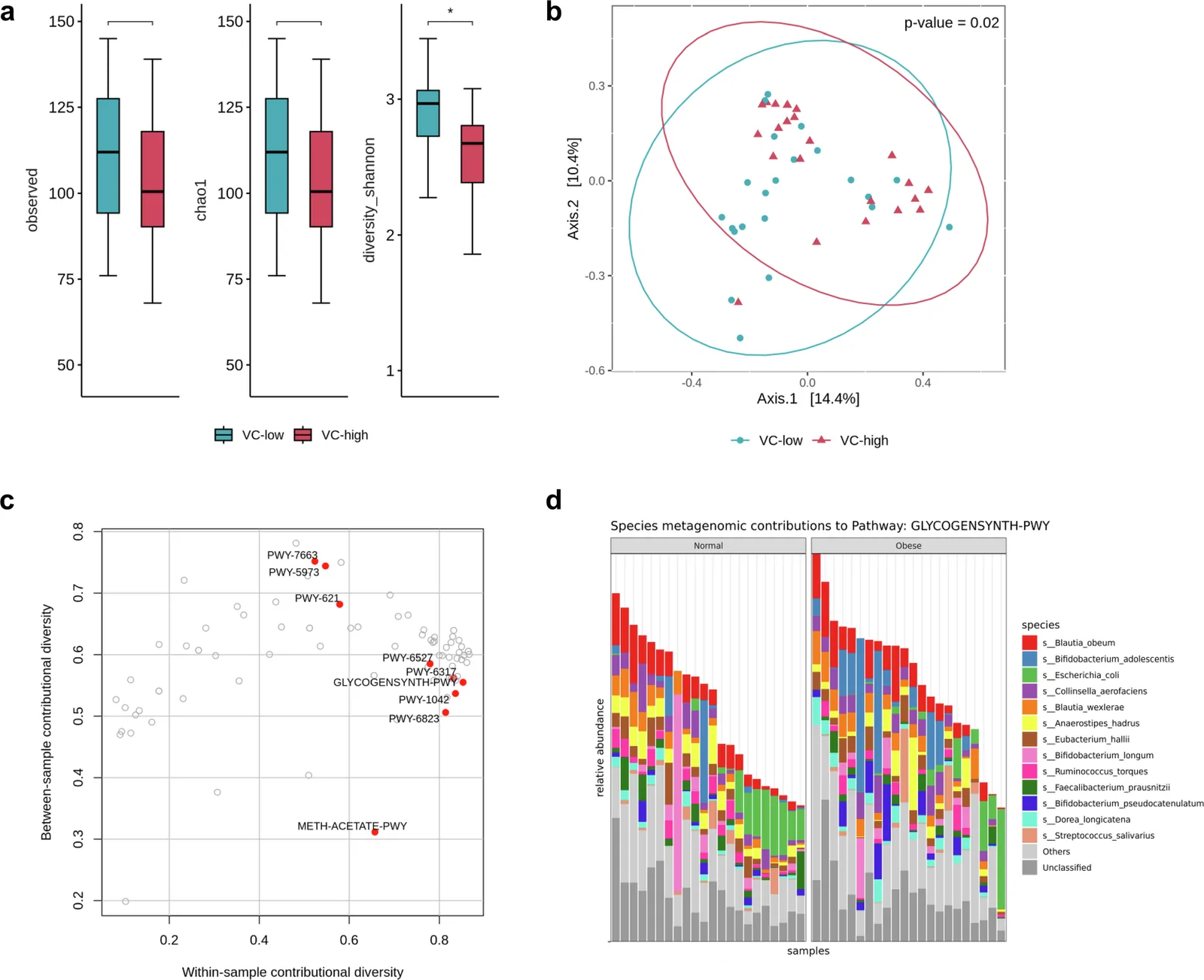
Metagenomic diversity of taxa and contributional diversity for core pathways. **a**, Observed Chao1 and Shannon indices for alpha diversity. An asterisk indicates a Wilcoxon rank-sum test *P* value less than 0.05. **b**, Jaccard distance for beta diversity plot for the visceral fat group with *P* values from PERMANOVA. **c**, Within- and between-participant contributional diversity of functions. Red dots represent pathways significantly correlated with *Dorea longicatena*. **d**, Diversity pattern of the pathway GLYCOGENSYNTH–PWY, which also correlated with visceral fat and PC1 among the correlated pathways in **c**.

We compared alpha and beta diversities between groups with high and low visceral fat (Fig. 5a, b). The high visceral fat group demonstrated reduced alpha diversity. Despite the limited sample size, a significant difference in the Shannon index, indicating evenness, was noted between the two groups (*P* < 0.05; Wilcoxon test). Beta diversity, assessed via a principal coordinates analysis plot using Jaccard distance, distinctly separated the two groups (*P* = 0.02; PERMANOVA).

In pathway analyses, we focused on pathways enhanced in at least 75% of participants and with 75% or more of their constituent taxa classified. This approach identified 85 core pathways from a total of 475 pathways^42^. *Dorea longicatena*, which was strongly associated with obesity and visceral fat, was significantly correlated with nine of these pathways (Supplementary Table 6).The contributional diversity plot (Fig. 5c, pathways marked as red dots) shows that the within-participant diversity values for these nine pathways consistently exceed 0.5, indicating a diverse microbial contribution to their activation, particularly activation of the GLYCOGENSYNTH–PWY (Fig. 5d) and other pathways (Supplementary Fig. 3). Among these pathways, only GLYCOGENSYNTH–PWY showed correlations with both PC1 and visceral fat (*P* for PC1 = 0.049, *P* for visceral fat = 0.097), exhibiting high within-participant contributional diversity but low between-participant diversity (Supplementary Table 7). *D. longicatena* showed a significantly positive correlation with this pathway (Supplementary Fig. 4).

Further analysis of taxa contributing to the nine pathways revealed that *B. adolescentis*, *Bifidobacterium pseudocatenulatum* and *D. longicatena* were significant contributors with higher PC1 values (Fig. 5d and Supplementary Fig. 3). Specifically, the abundance of *B. adolescentis* and *D. longicatena* was significantly correlated with PC1, total fat, WC and visceral fat. Importantly, these correlations were consistent across both 16S rRNA sequencing and shotgun sequencing data, as demonstrated in Supplementary Table 8. Moreover, the GLYCOGENSYNTH–PWY identified in the MetaCyc database through HuMAnN 3.0 corresponds to KEGG module M00854-dlo00500 (glycogen biosynthesis, glucose-1 P → glycogen/starch) in the KEGG database, integrated into the pathway ko00500 (starch and sucrose metabolism). This pathway is utilized by *D. longicatena* VE303-06 for glycogen synthesis (Supplementary Fig. 5). The identification of *D. longicatena*, *B. adolescentis* and the enriched glycogen synthesis pathway provided candidate targets for functional validation. To determine whether these candidates possess a causal role in obesity, we conducted in vivo experiments using a mouse model.

### Oral administration of *D. longicatena* and *B. adolescentis* promotes adiposity and hepatic lipogenesis

**Fig. 6:**
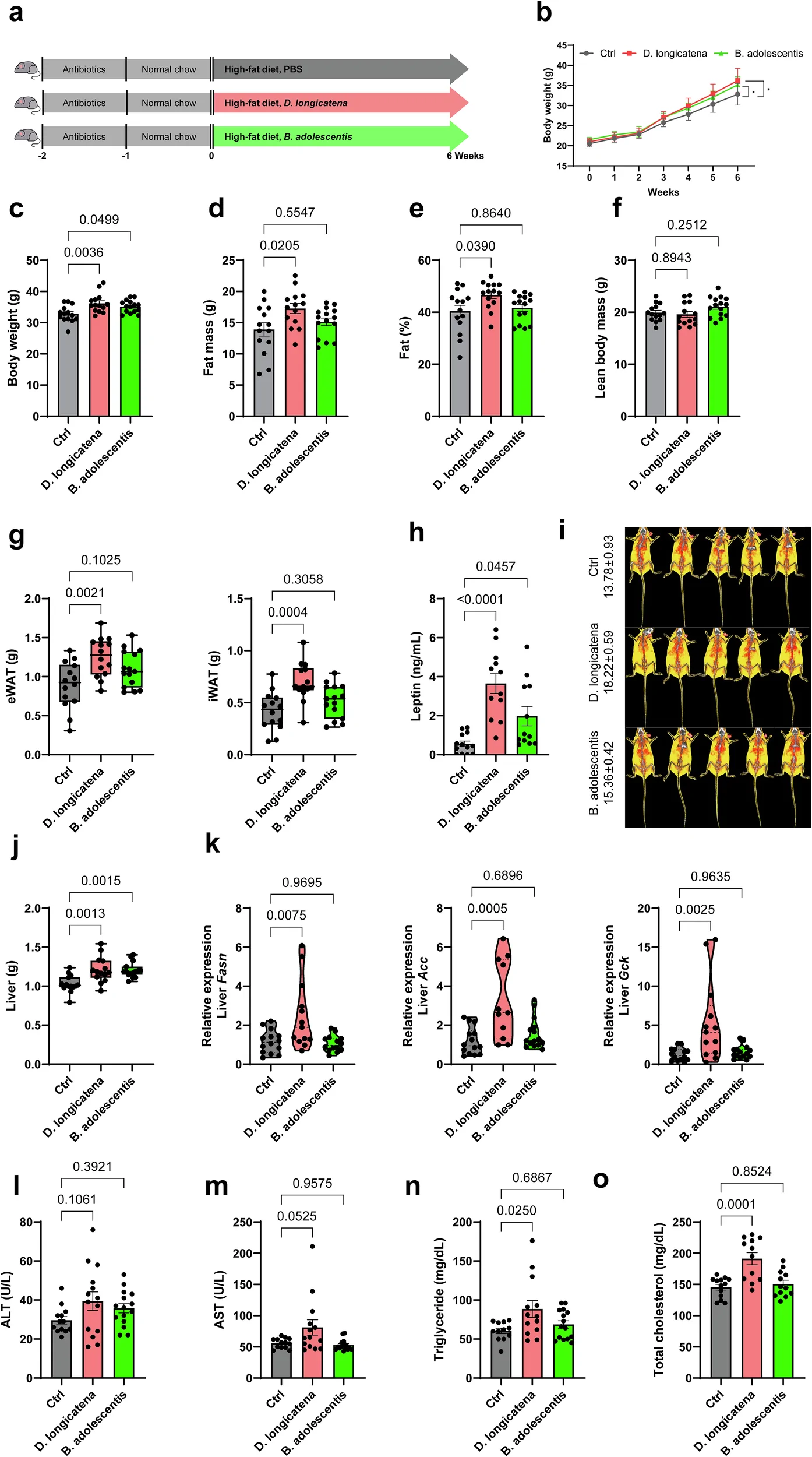
Effects of *Dorea longicatena* and *Bifidobacterium adolescentis* on obesity-related phenotypes in vivo. **a**, Schematic overview of the experimental design; mice received an antibiotic cocktail (ampicillin, neomycin and vancomycin) for 1 week for microbiota depletion, followed by 1 week of washout and 6 weeks of high-fat diet and with daily oral gavage of 10^8^ CFU or *D. longicatena*, *B. adolescentis* or PBS (Ctrl) (*n* = 14–15). **b**,**c**, Weekly body weight changes (**b**) and final body weight (**c**). **d**–**f**, Body composition analysis via DXA; fat mass (**d**), fat percentage (**e**) and lean body mass (**f**). **g**, Weights of eWAT and iWAT collected at euthanasia. **h**, Serum leptin levels. **i**, Representative fat composition (DXA) images of mice from each group. **j**, Liver weight at euthanasia. **k**, Relative mRNA expression of lipogenic genes (*Fasn*, *Acc* and *Gck*) in the liver. **l**–**o**, Serum metabolic parameters: ALT (**l**), AST (**m**), TG (**n**) and total cholesterol (**o**) levels. Statistical significance was determined by one-way analysis of variance (ANOVA) followed by Tukey’s post-hoc test; *P* values are indicated above the bars.

To investigate whether the identified candidate taxa directly induce obesity, we utilized a diet-induced obesity mouse model. Before microbial administration, mice were treated with an antibiotic cocktail for 1 week to deplete the endogenous microbiota. Following a washout period, mice were fed a high-fat diet supplemented with daily oral gavages of 10^8^ CFU of *D. longicatena* or *B. adolescentis* (Fig. 6a). Mice treated with either strain exhibited significantly higher body weight gain compared with the control group starting from week 3 to 4 (Fig. 6b, c). Dual-energy X-ray absorptiometry (DXA) analysis and tissue weighing confirmed that in the *D. longicatena* group, this weight gain was primarily driven by increased fat mass and fat percentage, while there was no difference in lean body mass (Fig. 6d–f, i). Specifically, the weights of visceral fat depots, including eWAT and iWAT, were significantly elevated in the *D. longicatena*-treated mice (Fig. 6g). Serum leptin levels were also markedly increased in both *D. longicatena* and *B. adolescentis* groups compared with control mice (Fig. 6h). Furthermore, liver weight significantly increased in both treatment groups, suggesting potential hepatic steatosis (Fig. 6j). To elucidate the underlying molecular mechanism, we analyzed the expression of lipogenic genes in the liver and pro-inflammatory gene in the eWAT. Mice colonized with *D. longicatena* showed a significant upregulation of *Fasn*, *Acc* and *Gck*, indicating that this taxon promotes obesity by stimulating the hepatic lipogenesis pathway (Fig. 6k). In addition, serum alanine aminotransferase (ALT), aspartate aminotransferase (AST), TG and total cholesterol of *D. longicatena*-treated mice showed increased tendency compared with control groups (Fig. 6l–o), it implies that these obesity-related taxa induce a metabolic obesity phenotype. These observations highlight the potential role of these microbial taxa in modulating host metabolism and contributing to obesity-related phenotypes. To verify metabolic association of candidate taxa, we performed LEfSe analysis with Korean clinical data. LEfSe plots demonstrated that certain microbial taxa, including *D. longicatena* and *B. adolescentis*, were positively associated with high-risk metabolic traits (Supplementary Fig. 6). Specifically, *D. longicatena* was correlated with risky WC, low HDL and high TG (Supplementary Fig. 6a,d,g). The enrichment of *D. longicatena* and *B. adolescentis* in high-risk metabolic groups underscores their potential role in promoting obesity-related phenotypes. Thus, oral administration of *D. longicatena* and *B. adolescentis* triggers significant weight gain and hepatic fat accumulation, with *D. longicatena* specifically driving potent upregulation of hepatic lipogenic genes and systemic metabolic dysfunction.

### Shift in microbial ecology and ecological network clustering

**Fig. 7:**
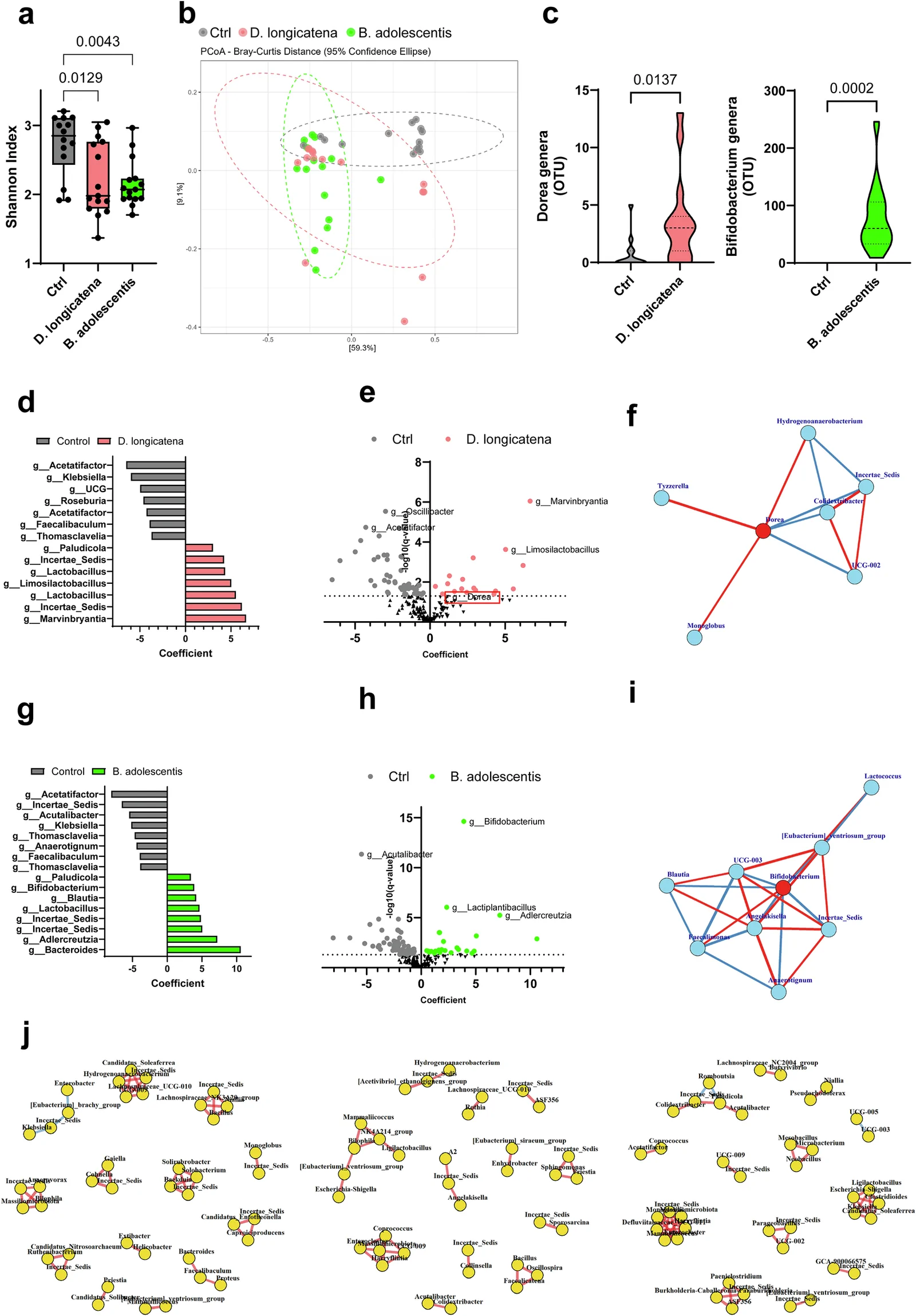
Metagenomic profiling of microbial taxa of diet-induced mouse model. **a**, Alpha diversity (Shannon index) comparing control, *D. longicatena* and *B. adolescentis* groups. **b**, PCoA plot of beta diversity using Bray–Curtis distance (95% confidence ellipses). **c**, Validation of colonization efficiency for *Dorea* and *Bifidobacterium* genera based on OTU counts; statistical significance was analyzed using a two-tailed Student’s *t*-test. **d**, **e**, Linear discriminant analysis (LDA) effect size (LEfSe) and volcano plots identifying differentially abundant taxa in *D. longicatena*-treated mice compared with the control. **f**, Ego-networks for *D. longicatena*, where red and blue lines indicate synergistic and competitive interactions, respectively. **g**, **h**, LDA (**g**) and volcano plots (**h**) identifying differentially abundant taxa in *B. adolescentis*-treated mice compared with the control. **i**, Ego-networks for *B. adolescentis*. **j**, Global microbial interaction networks for each experimental group, visualizing changes in community clustering, hub species and overall connectivity.

Next, we analyzed fecal microbial composition to elucidate microbial ecological alteration. Metagenomic analysis revealed that the administration of *D. longicatena* and *B. adolescentis* reshaped the gut microbial landscape. Both treated groups exhibited a marked reduction in alpha diversity, as measured by the Shannon index, which was observed in clinical cohort (Figs. 5a and 7a). PCoA based on Bray–Curtis distance showed distinct clustering of the *D. longicatena* and *B. adolescentis* groups away from the control group, confirming successful colonization and community-wide shifts (Fig. 7b). The successful enrichment of the target genera was further validated by the increased OTU counts of *Dorea* and *Bifidobacterium* in their respective groups (Fig. 7c). In addition, differential abundance analysis identified specific taxa that were enriched or depleted following colonization (Fig. 7d, e, g, h). To understand how these strains interact within the ecosystem, we constructed microbial ego-networks. *D. longicatena* showed synergistic links with *Tyzzerella* and *Monoglobus*, while *B. adolescentis* exhibited a strong synergistic cluster with the *[Eubacterium] ventriosum* group and *Lactococcus* (Fig. 7f, i). The global network clusters for each group demonstrated that the administration of these candidate strains leads to a more interconnected and obesity-associated microbial community structure compared with the control group (Fig. 7j). Collectively, exogenous administration of *D. longicatena* and *B. adolescentis* successfully remodels the gut ecosystem into a low-diversity, highly interconnected state characterized by the enrichment of obesity-associated synergistic microbial networks.

## Discussion

This study presents an extensive investigation into the relationship between obesity-related clinical variables and gut microbiota in a large cohort of healthy Koreans. We focused on phenotypes such as visceral and total fat, SBP, DBP, insulin and HDL cholesterol, which demonstrated significant correlations with the gut microbiota. Although the association between obesity and gut microbiota is well established^43^, identification of specific microbial biomarkers has been limited^44,45^. Our research uncovers potential microbial biomarkers related to obesity and reinforces the concept that obesity, considered the second most serious health threat in the absence of overt disease, is fundamentally associated with the gut microbiome.

Among the clinical variables studied, visceral fat showed a more consistent and significant association with gut microbiota than BMI, total fat or WC. Visceral fat is critically associated with the onset of insulin resistance, type 2 diabetes, elevated TG and cardiovascular disease^20^. It secretes pro-inflammatory cytokines and adipokines that disrupt insulin signaling and promote insulin resistance. In addition, its metabolic activity is a key contributor to increased TG production and accumulation in the liver^46^. Recent studies have highlighted a relationship between the composition of the gut microbiome and both obesity and visceral fat amount in humans. Specific microbial groups, including *Proteobacteria*, *Actinobacteria* and *Verrucomicrobia*, have been associated with obesity^3,8,47,48^. The mechanisms through which the microbiota influences obesity are multifaceted, ranging from enhanced dietary fiber fermentation, which leads to increased energy absorption, to the suppression of a liver enzyme critical for fat metabolism regulation^8^.

Our findings indicate a reduction in alpha diversity, reflecting both species richness and evenness, in individuals with higher visceral fat levels (Fig. 5a). Our observation of low alpha diversity paired with heightened activity in the microbial glycogen synthesis pathway uniquely correlates with elevated visceral adiposity. Indeed, at the host physiological level, such a correlation aligns with the understanding that excessive glycogen accumulation is associated with inflammation, insulin resistance, and increased glycogen storage in adipose tissue^49^. Although high-fat diets generally reduce glycogen accumulation in the liver over time, they also lead to an increase in the amount of abdominal adipose tissue^50^. In obese mice, glycogen synthesis is reduced in muscle tissue but overall increased in various other tissues^51^. Combining these observations with our research findings suggests that the gut-microbiota-induced increase in glycogen synthesis occurs distinctively within abdominal adipose tissue.

We identified microbiota significantly and specifically associated with obesity and the underlying mechanisms. However, the four enterotypes identified in this study should be interpreted as representative microbial patterns that reflect the continuous distribution of gut microbiota in a large population, rather than as strictly discrete biological entities. Also, the two-dimensional projection may not sufficiently visualize the clusters’ separation that exists in a higher-dimensional space^52^. The positive association of *D. longicatena* with obesity has not been conclusively established owing to a lack of clarity on its mechanistic details^53,54^. Similarly, the relationship between *B. adolescentis* and obesity and visceral fat accumulation lacks a clear consensus. Chen et al. reported *B. adolescentis* alleviating visceral fat accumulation in a mouse model^55^, whereas Wang et al. reported conflicting results, showing the potential for both induction and inhibition of obesity by the same species^56^. Similarly, Ignacio et al. observed a higher prevalence of *B. adolescentis* in obese and overweight children, with results variably correlating with BMI^57^. However, our study revealed that the prominence of the glycogen synthesis pathway is positively associated with the increased presence of *D. longicatena* or *B. adolescentis*, suggesting a potential link between gut microbiota in promoting obesity and high visceral fat levels. *D. longicatena* abundance is correlated with the glycogen biosynthesis I pathway (from ADP-D-glucose)^58^, which converts glucose into glycogen in the liver and muscles, with surplus glucose stored as abdominal visceral fat^59^. Our findings further emphasize the critical role of this pathway in supporting obesity, especially by enhancing visceral fat accumulation. The increase in *D. longicatena* abundance seems to share similar metabolic characteristics with insulin-mediated glucose utilization, specifically by shunting glucose toward storage pathways and potentially impeding lipid mobilization, thus impeding the breakdown of fat. This can lead to fat accumulation, high visceral fat and ultimately obesity. Furthermore, our findings suggest that this mechanism may stimulate glycogen synthesis and promote TG production from visceral fat and insulin secretion, which showed a positive correlation with *D. longicatena* and other candidate taxa. *Dorea longicatena*, recognized in both North American and Korean standard reference lists, is prevalent across diverse populations (Fig. 4), highlighting its global significance in obesity management beyond racial and ethnic boundaries. Its widespread prevalence may facilitate streamlined management strategies, ensuring a unified approach to obesity treatment irrespective of ethnicity.

*B. adolescentis* was one of the most prevalent species among Koreans, highlighting its distinct association with obesity within this population. This bacterium has been recognized for its role in glycogen synthesis in obese individuals and its significant contribution to the production of gamma-aminobutyric acid (GABA)^60^. Given *B. adolescentis* produces GABA and that glycogen is involved in GABA synthesis^61^, there may be a correlation between *B. adolescentis* and the glycogen synthesis pathway. Nevertheless, the precise influence of this bacterium on obesity remains uncertain. Therefore, although it shows promise for targeted interventions in the Korean population, further research is essential to fully understand its impact.

One major limitation of this study is that the biochemical mechanism bridging intracellular bacterial glycogen synthesis and host hepatic lipogenesis remains to be fully elucidated. It is crucial to acknowledge that the enriched bacterial glycogen synthesis pathway and the upregulation of lipogenic genes (*Fasn*, *Acc* and *Gck*) observed in our mouse model represent two independent associative findings. While *D. longicatena* administration clearly alters host lipid metabolic profiles, we cannot definitively prove how an intracellular bacterial storage process directly triggers host physiology. These findings should therefore be interpreted as parallel insights that together generate a compelling hypothesis, warranting direct measurement of cecal or fecal bacterial glycogen content in future mechanistic studies. Nevertheless, the pivotal finding of this study is that *D. longicatena* and *B. adolescentis* are not merely associated with obesity but act as key contributors that exacerbate or modulate visceral fat accumulation. While previous reports on these taxa have been inconsistent, our current in vivo data clearly demonstrates that their administration triggers significant weight gain and visceral fat expansion (eWAT and iWAT). The molecular mechanism underlying this induction involves the stimulation of hepatic lipogenesis. Specifically, mice colonized with *D. longicatena* exhibited a significant upregulation of *Fasn*, *Acc* and *Gck* in the liver (Fig. 6k). This mirrors a hypermetabolic state where surplus glucose is shunted toward fatty acid synthesis, thereby promoting fat accumulation and systemic metabolic dysfunction. In our mouse model, the administration of these candidate strains successfully remodeled the gut ecosystem into a low-diversity state. Ego-network analysis revealed that these strains establish synergistic clusters with obesity-associated taxa, such as the *[Eubacterium] ventriosum* group and *Lactococcus*, thereby creating a highly interconnected microbial community that facilitates energy harvest (Fig. 7).

Another limitation is the lack of research on patients with metabolic syndrome. Although studying healthy individuals enables the observation of gut microbiomes without the confounding factors introduced by metabolic syndrome, examining a metabolically compromised population could offer insights into potential differences in these associations. Including patients with metabolic syndrome and conducting sensitivity analyses in this group could help to identify variations and strengthen the findings. Future studies should aim to include diverse cohorts, particularly those with metabolic syndrome, to validate and extend these findings, providing a more comprehensive understanding of the host–microbiome relationship and its role in visceral fat accumulation.

**Fig. 8:**
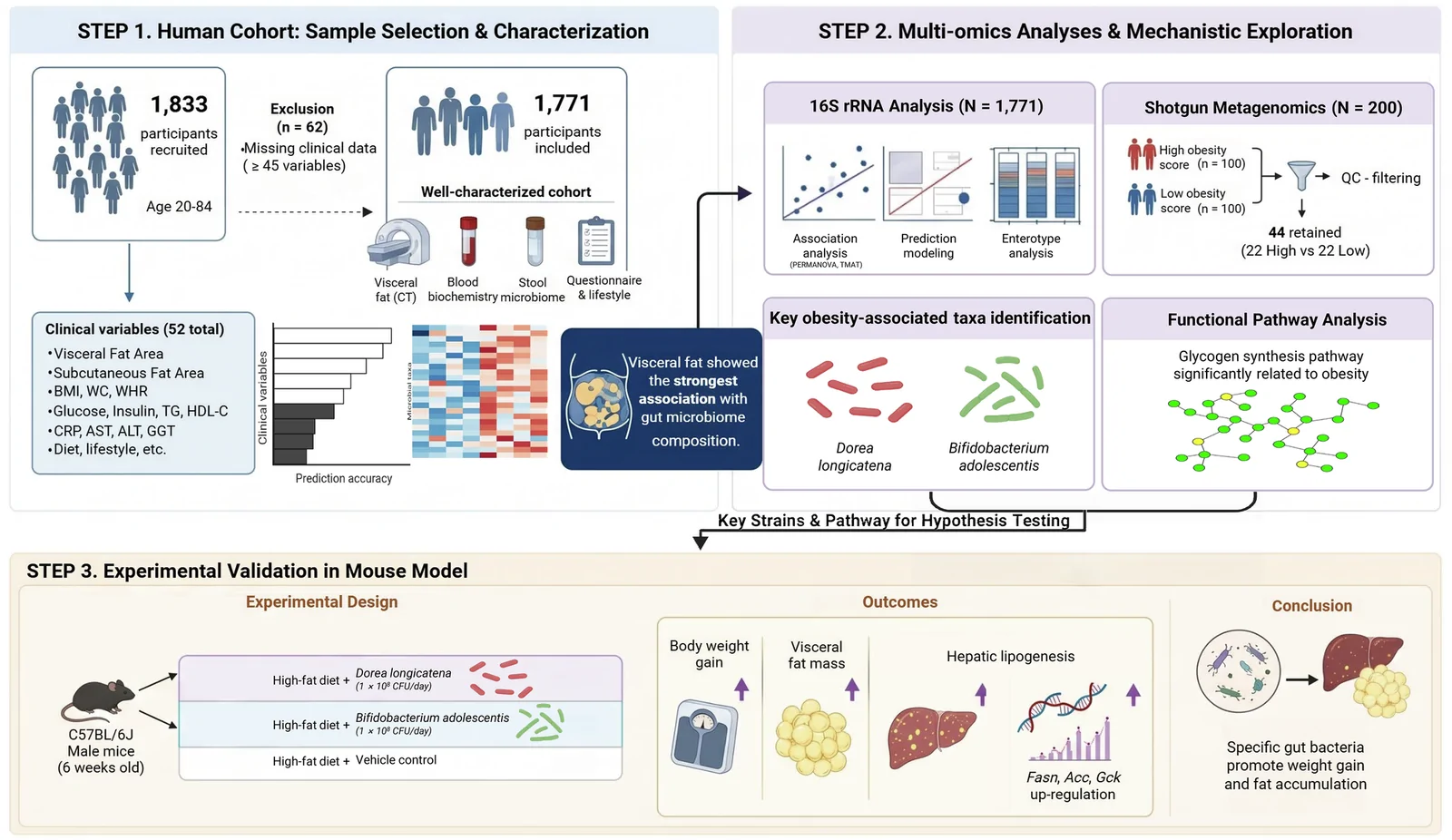
Schematic overview of the study: from human cohort screening to mechanistic validation in mice. Schematic overview of cohort selection, microbiome analyses, candidate identification and experimental validation workflow used in this study.

In conclusion, this Article illustrates the link between certain bacteria and obesity, suggesting that understanding microbiota–obesity interactions could enhance treatment predictions and outcomes (Fig. 8). However, this research introduces innovative approaches for obesity management and preventing metabolic disorders. Although these findings cannot be directly applied in clinical settings without further validation, they provide insights for designing future therapies. These could include personalized treatments that customize healthcare strategies to individual microbiota profiles, thereby effectively addressing the complexities of obesity.

## Data and materials availability

The dataset analyzed during the current study is available in the BioProject repository ID: PRJNA1113191 (https://www.ncbi.nlm.nih.gov/sra/PRJNA1113191). Accession numbers: SRR29261648–SRR29261686, SRR29253385–SRR29253810, SRR29179565–SRR29179913 for 16S rRNA sequencing data; SRR33210144–SRR33210182 for shotgun sequencing data. Additional data for reanalysis can be requested from the lead author upon contact.

## Supplementary information

Supplementary Information

Supplementary Table 5

Supplementary Table 6

Supplementary Table 7

Supplementary Table 8
