# Supplementary material for: Linking visceral fat accumulation to gut microbiota: key bacterial taxa and their roles in the glycogen synthesis pathway
Source: Exp Mol Med. 2026 Sep 10;58(9):3113–26. doi: 10.1038/s12276-026-01828-7 (PMC13633279; doi:10.1038/s12276-026-01828-7)
Supplement: Supplementary file 1 [file 12276_2026_1828_MOESM1_ESM.pdf]

<Supplementary data for *Experimental & Molecular Medicine*>

Linking visceral fat accumulation to gut microbiota: Key bacterial taxa and their roles in the glycogen synthesis pathway

Nam-Eun Kim[1†], Beomki Cho[2†], Yunjae Kim[2], Gihyeon Kim[3,4], Ga-Eun Yie[5], Haeon Kim[2], Jaesung Park[2], Hyun Kim[2], Chanyoung Jeong[2], Minji Lee[2], Yanghyun Joo[2], Sua Lim[2], Sihyeon Park[2], Yeji Hong[2], Kyungchan Min[2], Belong Cho[7,8], Jae Moon Yun[7,8], Eun Sung Jung[9], Yosep Ji[9], Seongbeom Cho[12], Je Kyung Seong[13,14], Hansoo Park[2,6*], Jin-Ho Park[7,8*], Sungho Won[1,5,10,11*]

[1] Institute of Health and Environment, Seoul National University, Seoul, Republic of Korea

[2] Department of Biomedical Science and Engineering, Gwangju Institute of Science and Technology (GIST), Gwangju, Republic of Korea

[3] Genomic Medicine Institute, Medical Research Center, Seoul National University, Seoul, Republic of Korea

[4] Department of Biomedical Sciences, Seoul National University College of Medicine, Seoul, Republic of Korea

[5] Interdisciplinary Program in Bioinformatics, Seoul National University, Republic of Korea

[6] Genome and Company, Gyeonggi-do, Republic of Korea

[7] Department of Family Medicine, Seoul National University Hospital, Seoul, Republic of Korea

[8] Department of Family Medicine, Seoul National University College of Medicine, Seoul, Republic of Korea

[9] HEM Pharma, Gyeonggi-do, Republic of Korea

[10] RexSoft Corp, Seoul, Republic of Korea

[11] Department of Public Health Sciences, Seoul National University, Seoul, Republic of Korea

[12]College of Veterinary Medicine and Research Institute for Veterinary Science, Seoul National University, Seoul, Republic of Korea

[13]Laboratory of Developmental Biology and Genomics, Research Institute for Veterinary Science, and BK21 PLUS Program for Creative Veterinary Science Research, College of Veterinary Medicine, Seoul National University, Seoul, Republic of Korea

[14]Korea Model Animal Priority Center, Seoul National University, Seoul, Republic of Korea

[†]These authors contributed equally to this study.

[*]Corresponding authors

Hansoo Park

Department of Biomedical Science and Engineering, Gwangju Institute of Science and Technology, Gwangju, 61005, South Korea

Tel: +82-62-715-5414, E-mail: hspark27@gist.ac.kr

Jin-Ho Park

Department of Family Medicine, Seoul National University College of Medicine and Seoul National University Hospital, Seoul, 03080, Republic of Korea

E-mail: pjhn@snu.ac.kr

Sungho Won

Department of Public Health Sciences, Seoul National University, Seoul, 151-742, Republic of Korea

Tel: +82-2-880-2714, E-mail: won1@snu.ac.kr

Supplementary material

Supplementary Fig. 1. Comparison of bacterial taxa composition between 16S rRNA and shotgun metagenome data.

Supplementary Fig. 2. Network analysis of the prevalent Korean gut microbiota and related host metabolites.

Supplementary Fig. 3. Contribution plot illustrating significantly correlated pathways with *Dorea longicatena.*

Supplementary Fig. 4. Scatter plot of *Dorea longicatena* and GLYCOGENSYNTH-PWY.

Supplementary Fig. 5. KEGG pathway map (ko00500 - Starch and sucrose metabolism) matched with GLYCOGENSYNTH-PWY from MetaCyc.

Supplementary Fig. 6. Linear discriminant analysis (LDA) of microbial taxa associated with clinical metabolic traits.

Supplementary Table 1. Baseline characteristics of clinical variables.

Supplementary Table 2. Results of statistical analysis evaluating the impact of Enterotypes on clinical variables.

Supplementary Table 3. Comparison of median values of clinical variables among the four Enterotypes.

Supplementary Table 4. Association test using TMAT between principal components (PCs) and 16S rRNA bacterial taxa.

Supplementary Table 5. Comparison of median values of 16S rRNA bacterial taxa among the four Enterotypes.

Supplementary Table 6. Association analyses between *Dorea longicatena* and pathways by linear regression with clinical variables.

Supplementary Table 7. Association test using linear regression between clinical variables and pathways.

Supplementary Table 8. Association analyses by linear regression with clinical variables.

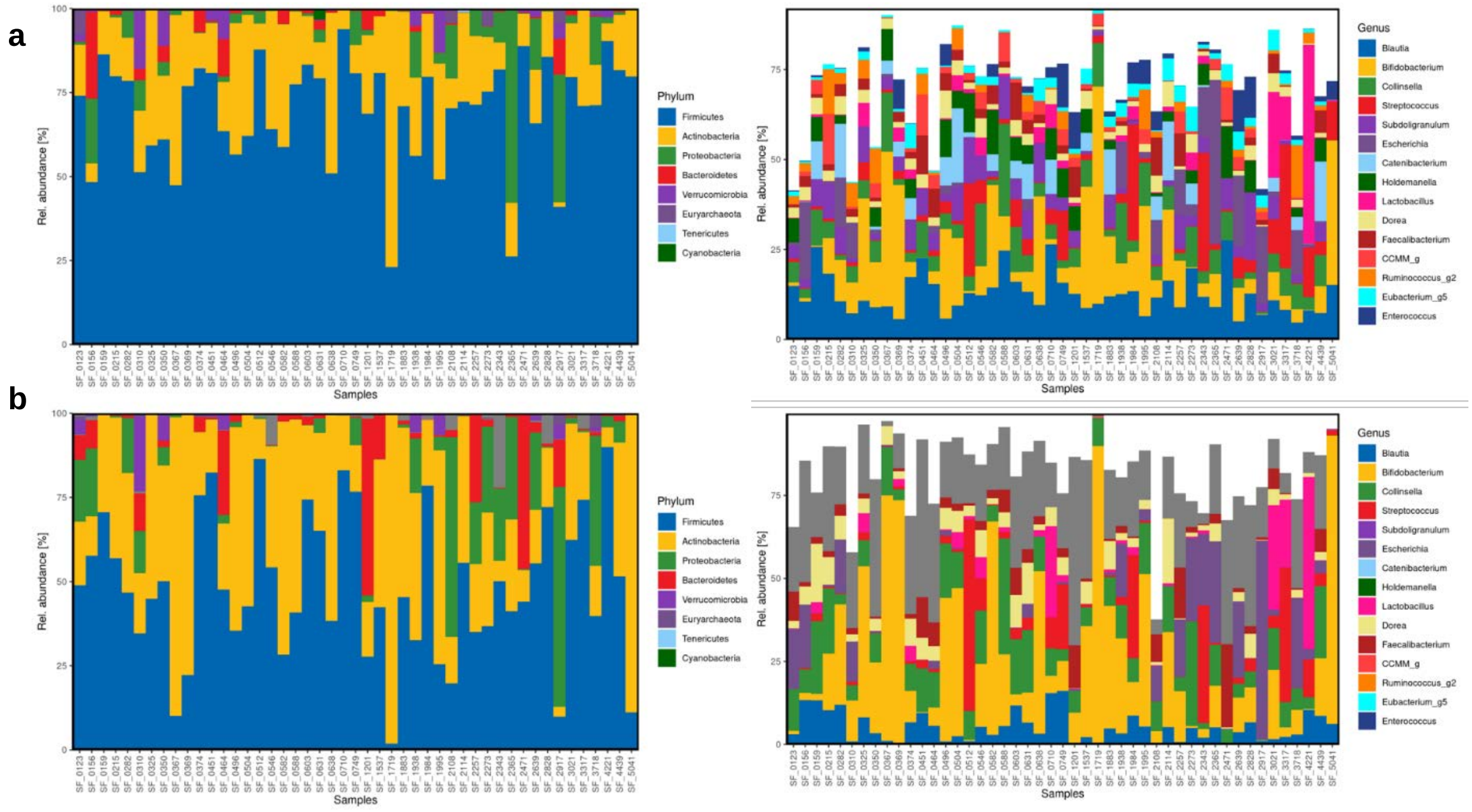

Supplementary Fig. 1. Comparison of bacterial taxa composition between 16S rRNA and shotgun metagenome data. a Phylum-level composition. **b** Genus-level composition. The upper and lower plots represent the 16S rRNA composition and the metagenome composition, respectively.

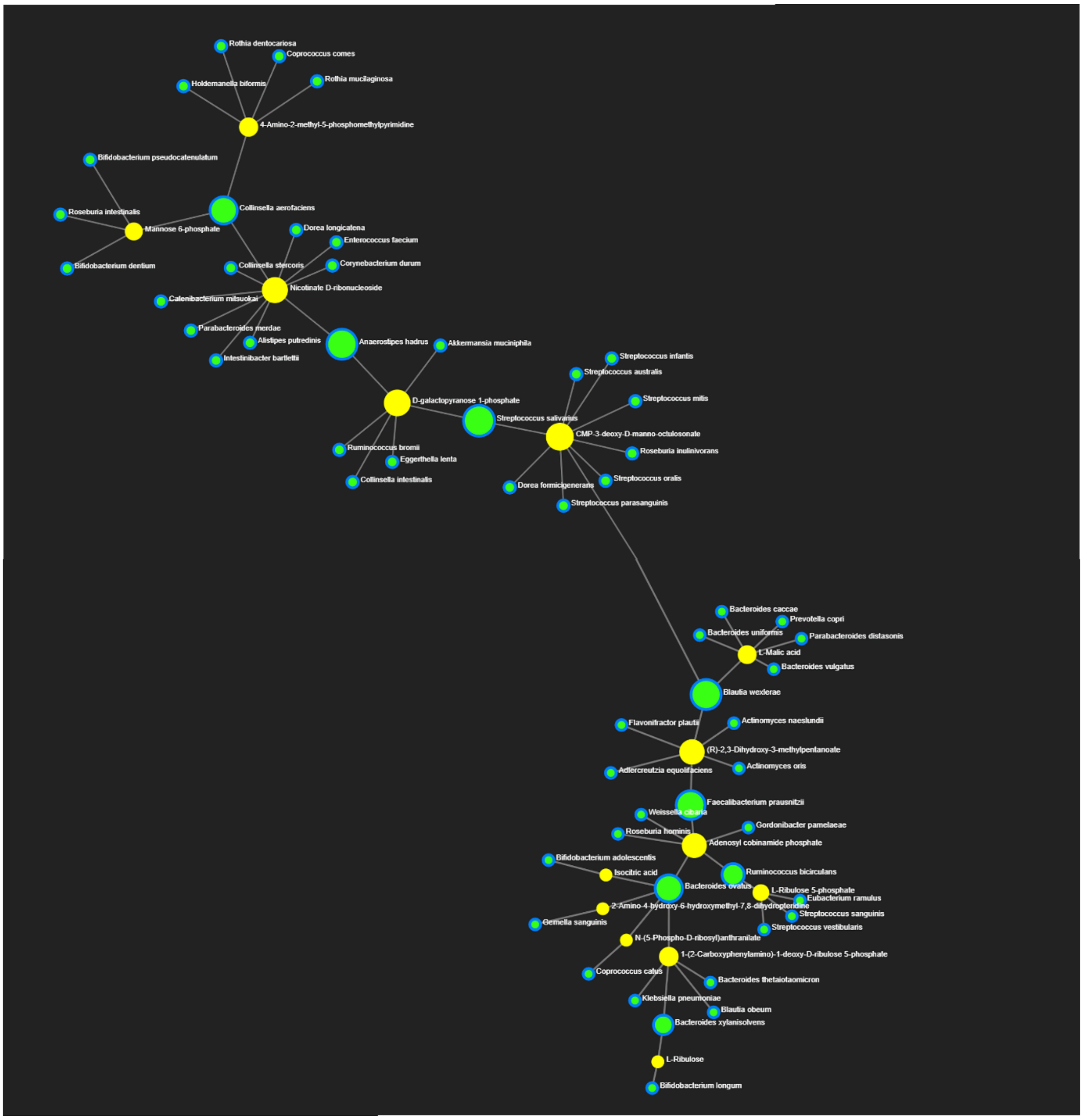

Supplementary Fig. 2. Network analysis of the prevalent Korean gut microbiota and related host metabolites. The network illustrates the functional connections among a subset of the 91 prevalent species that show significant metabolic potential (potential score < 0.8) using AGORA GEMS and OmicsNet.

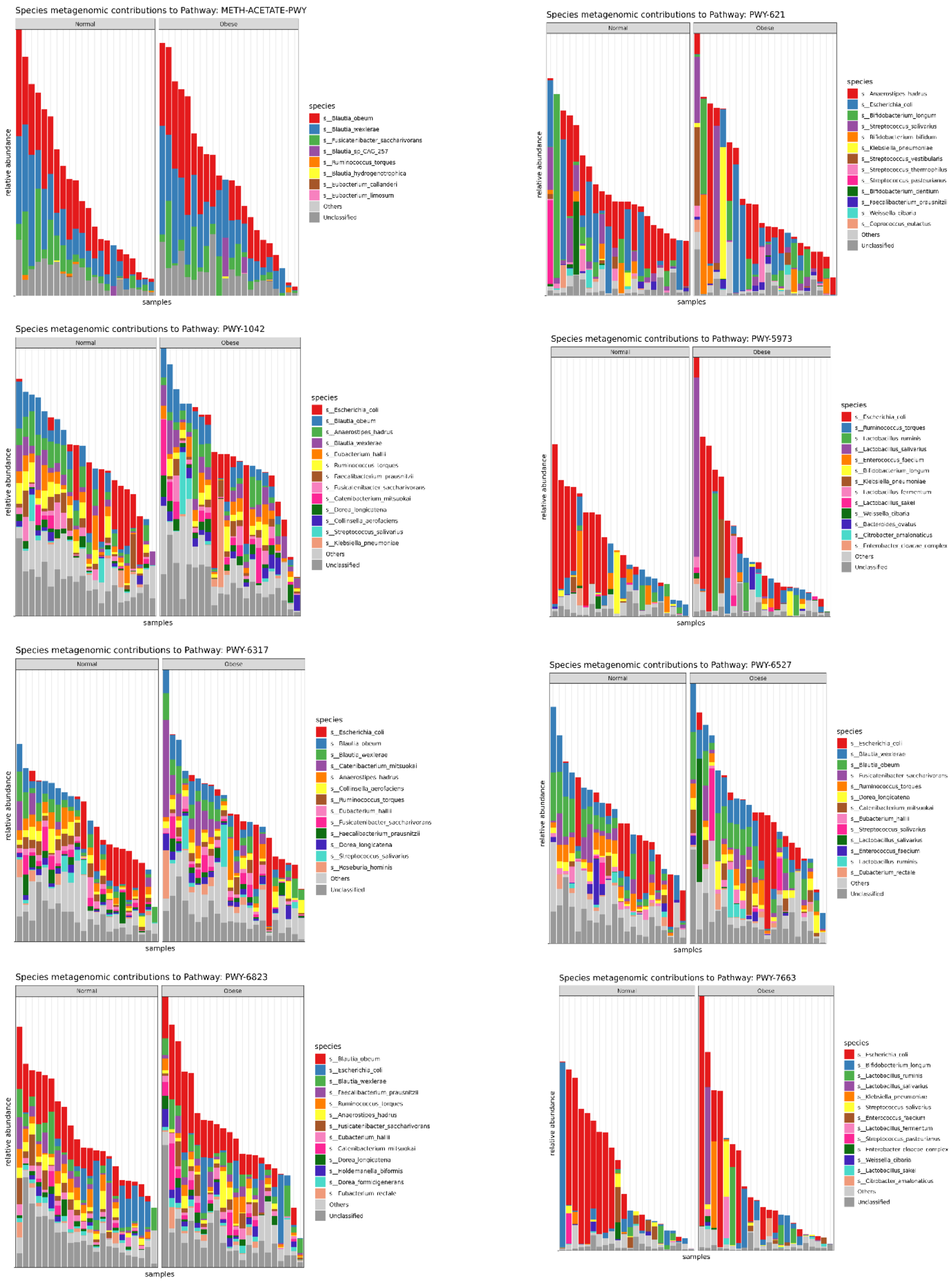

Supplementary Fig. 3. Contribution plot illustrating significantly correlated pathways with *Dorea longicatena*. The pathways include the following: METH-ACETATE-PWY, PWY-7663, PWY-5973, PWY-621, PWY-6527, PWY-6823, PWY-1042, and PWY-6317.

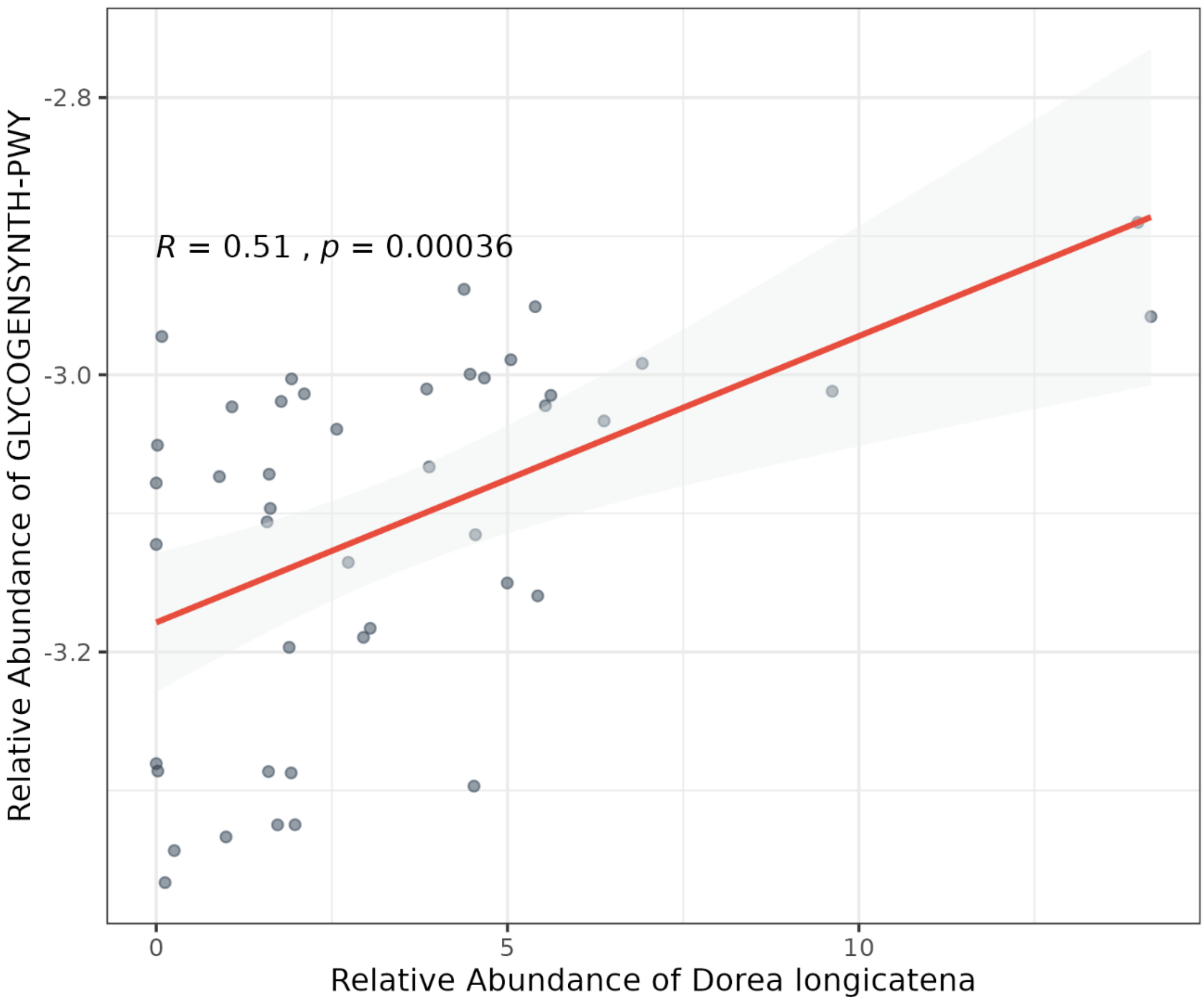

Supplementary Fig. 4. Scatter plot of *Dorea longicatena* and GLYCOGENSYNTH-PWY. Correlation between the relative abundance of *D. longicatena* and its metagenomic contribution to the GLYCOGENSYNTH-PWY.

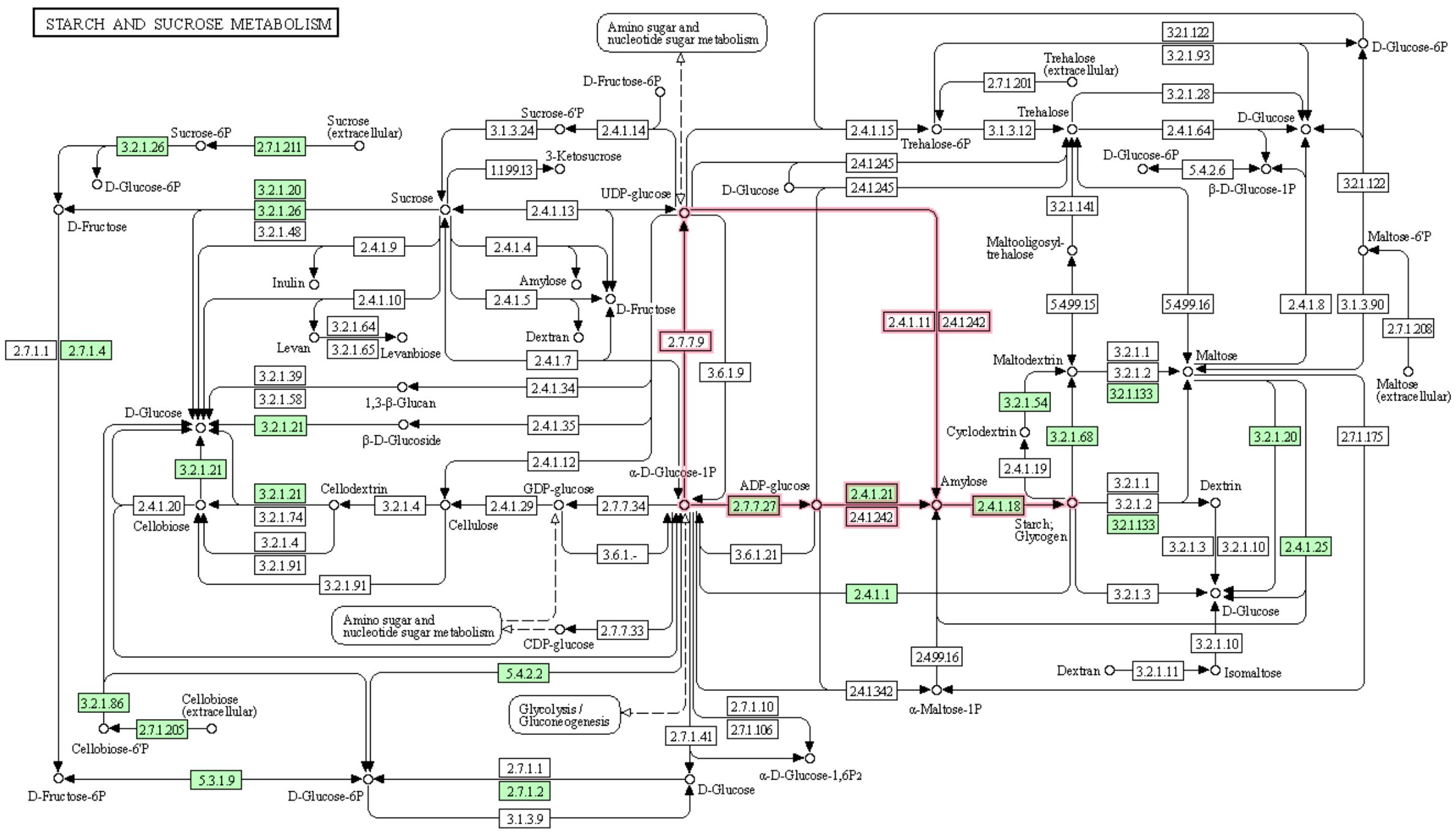

Supplementary Fig. 5. KEGG pathway map (ko00500 - Starch and sucrose metabolism) matched with GLYCOGENSYNTH-PWY from MetaCyc. The pathway highlighted using pink lines corresponds to KEGG module M00854-dlo00500 (Glycogen biosynthesis, glucose-1 P → glycogen/starch) of the *Dorea longicatena* VE303-06.

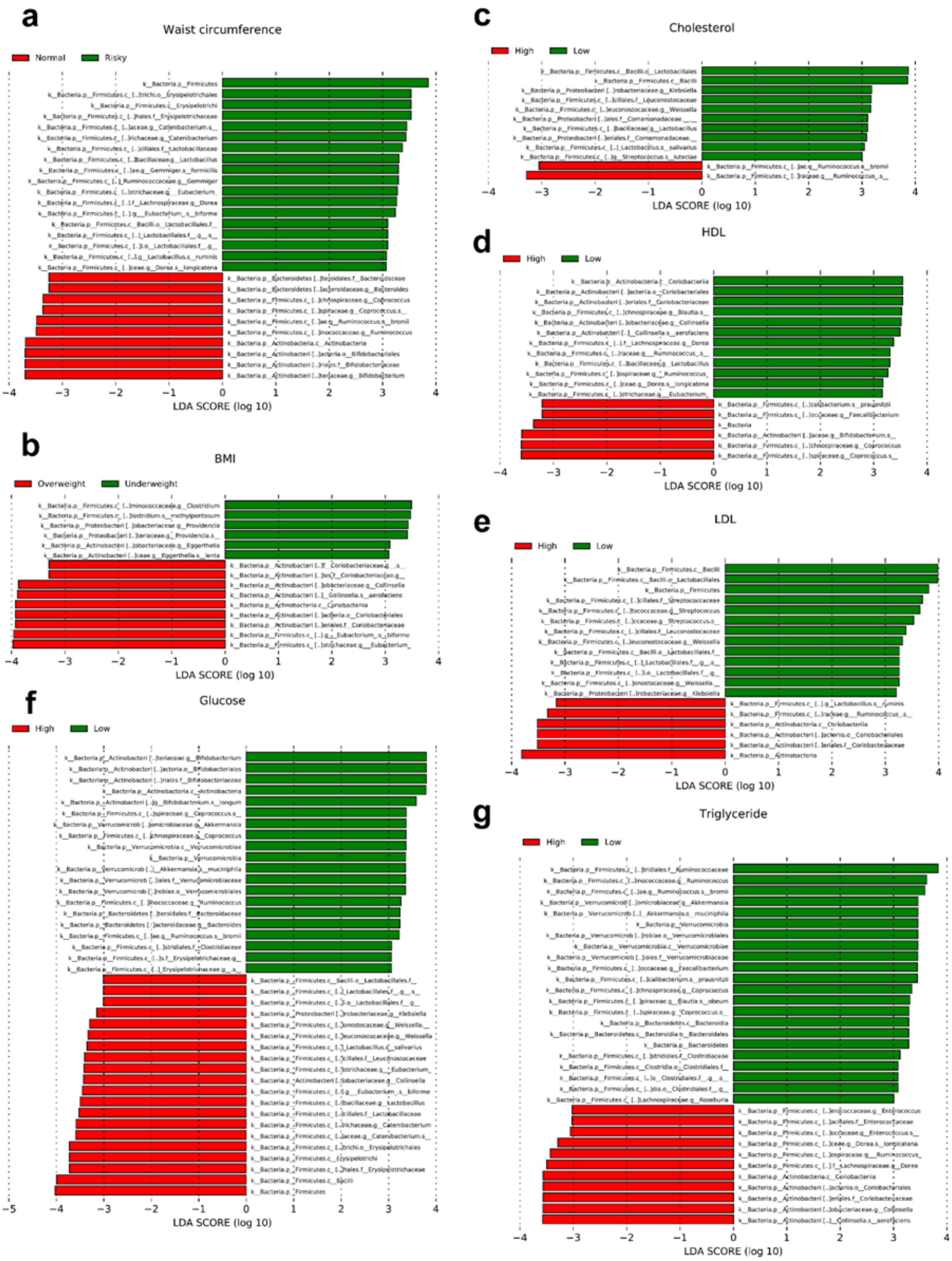

Supplementary Fig. 6. Linear discriminant analysis (LDA) of microbial taxa associated with clinical metabolic traits. a-g LDA effect size (LEfSe) plot showing microbial taxa enriched in high-risk versus low-risk groups for various clinical metabolic parameters: waist circumference (**a**), body mass index (**b**), cholesterol (**c**), high density lipoprotein (HDL; **d**), low density lipoprotein (LDL; **e**), glucose (**f**), and triglyceride (**g**).

Level	Overall
**n**	**1771**
Age (mean (SD))	58.05 (11.43)
Sex (%) F	800 (45.2)
Sex (%) M	971 (54.8)
BMI (mean (SD))	23.85 (3.09)
Waist circumference (mean (SD))	85.42 (9.25)
Heart rate (mean (SD))	72.19 (11.38)
Diastolic BP (mean (SD))	74.38 (10.32)
Systolic BP (mean (SD))	123.23 (15.62)
Percent body fat (mean (SD))	26.08 (6.28)
Eye press (Right) (mean (SD))	15.54 (2.42)
Eye press (Left) (mean (SD))	15.99 (2.38)
Mean DEXA (L1–L4) (mean (SD))	-0.07 (1.08)
DEXA (Femur neck) (mean (SD))	-0.29 (0.78)
Total fat (mean (SD))	287.82 (68.11)
Visceral fat (mean (SD))	126.64 (49.32)
Calcium (mean (SD))	9.34 (0.36)
Phosphorus (mean (SD))	3.45 (0.48)
Glucose (mean (SD))	99.23 (21.62)
BUN (mean (SD))	14.21 (3.82)
Uric acid (mean (SD))	5.53 (1.34)
Cholesterol (mean (SD))	196.89 (38.72)
Total protein (mean (SD))	7.36 (0.39)
Albumin (mean (SD))	4.43 (0.26)
Total bilirubin (mean (SD))	1.03 (0.40)
Alkaline phosphatase (mean (SD))	63.13 (20.10)
AST (mean (SD))	25.19 (10.43)
ALT (mean (SD))	26.12 (17.84)
GGT (mean (SD))	34.94 (38.24)
TFT (free T4) (mean (SD))	1.18 (0.16)
TFTTSH (mean (SD))	1.99 (4.25)
OH-Vitamin D3 (mean (SD))	25.04 (8.78)
Creatinine (mean (SD))	0.83 (0.18)
Na (mean (SD))	141.88 (1.87)
K (mean (SD))	4.08 (0.30)
Cl (mean (SD))	104.74 (3.32)
Total CO2 (mean (SD))	28.09 (2.74)

Triglyceride (mean (SD))	110.71 (64.64)
HDL cholesterol (mean (SD))	60.22 (16.36)
LDL cholesterol (mean (SD))	116.85 (35.48)
hsCRP (mean (SD))	0.12 (0.36)
ESR (mean (SD))	14.22 (12.21)
WBC (mean (SD))	5.70 (1.66)
RBC (mean (SD))	4.72 (0.50)
Hemoglobin (mean (SD))	14.44 (1.55)
Hematocrit (mean (SD))	43.58 (4.21)
MCV (mean (SD))	92.45 (4.05)
RDW (mean (SD))	12.64 (0.81)
Platelet (mean (SD))	242.90 (57.19)
HbA1c (mean (SD))	5.88 (0.76)
Insulin (mean (SD))	7.94 (2.43)
AFP (mean (SD))	3.50 (3.98)
CEA (mean (SD))	1.71 (1.15)
PSA (mean (SD))	1.42 (1.10)
CA 19-9 (mean (SD))	8.47 (10.47)
CA 125 (mean (SD))	14.61 (22.31)

Supplementary Table 1. Baseline characteristics of clinical variables.

Clinical variables	P	Adjusted P
GGT	3.25E-08	1.82E-06
Triglyceride	2.30E-05	0.001
Insulin	2.70E-05	0.001
Systolic BP	4.10E-05	0.002
Visceral fat	1.42E-04	0.007
WBC	0.001	0.028
Alkaline phosphatase	0.002	0.077
Glucose	0.002	0.093
Diastolic BP	0.002	0.099
Uric acid	0.002	0.099
BMI	0.003	0.130
hsCRP	0.004	0.167
Total fat	0.004	0.187
Percent body fat	0.004	0.188
Heart rate	0.006	0.233
Albumin	0.007	0.291
RBC	0.008	0.33
Waist circumference	0.01	0.368
Calcium	0.011	0.384
ALT	0.01	0.384
CA 125	0.012	0.412
HbA1c	0.014	0.489
DEXA (Femur neck)	0.016	0.519
CA 19-9	0.016	0.522
Hemoglobin	0.021	0.652
Phosphorus	0.033	0.964
Eye press (Right)	0.558	1
Eye press (Left)	0.165	1
Mean DEXA (L1~L4)	0.529	1
BUN	0.478	1
Cholesterol	0.473	1
Total protein	0.083	1
Total bilirubin	0.173	1
AST	0.254	1
TFT (free T4)	0.36	1
TFTTSH	0.911	1
OH-Vitamin D3	0.988	1
Creatinine	0.09	1
Na	0.457	1
K	0.324	1
Cl	0.755	1
Total CO2	0.519	1
HDL cholesterol	0.597	1
LDL cholesterol	0.041	1
ESR	0.183	1
Hematocrit	0.051	1
MCV	0.284	1
RDW	0.204	1
Platelet	0.805	1
AFP	0.536	1
CEA	0.234	1
PSA	0.12	1

Supplementary Table 2. Results of statistical analysis evaluating the impact of Enterotypes on clinical variables.

	Enterotype 1	Enterotype 2	Enterotype 3	Enterotype 4	ANOVA	
n	633	478	348	312	P	FDR
**Waist circumference**	4.44 [4.37;4.52]	4.45 [4.37;4.51]	4.43 [4.35;4.49]	4.48 [4.42;4.54]	2.41E-08	1.28E-06
**Heart rate**	2.05 [2.03;2.06]	2.05 [2.03;2.07]	2.05 [2.03;2.06]	2.05 [2.03;2.06]	0.000303	0.011214
**Diastolic BP**	27.91 [26.25;29.53]	28.18 [25.97;30.07]	27.36 [25.40;29.27]	28.46 [26.53;30.33]	3.84E-05	0.001638
**Systolic BP**	4.81 [4.72;4.90]	4.81 [4.72;4.89]	4.78 [4.69;4.87]	4.85 [4.74;4.92]	1.96E-06	9.78E-05
Percent body fat	32.94 [27.56;39.95]	33.57 [27.84;39.95]	33.50 [27.43;39.24]	32.39 [27.22;38.19]	0.49649	1
Eye press (Right)	15.34 [14.00;17.00]	15.00 [14.00;17.00]	15.00 [14.00;17.00]	15.48 [14.00;17.00]	0.73642	1
Eye press (Left)	2.77 [2.64;2.85]	2.77 [2.64;2.83]	2.77 [2.64;2.83]	2.77 [2.67;2.89]	0.48536	1
Mean Dexa (L1–L4)	-0.86 [-1.71;-0.02]	-1.05 [-1.61;-0.31]	-0.84 [-1.79;0.10]	-0.97 [-1.65;-0.22]	0.303	1
DEXA (Femur neck)	-0.20 [-0.64;0.11]	-0.22 [-0.72;0.09]	-0.25 [-0.72;0.00]	-0.20 [-0.64;0.07]	0.27695	1
**Total fat**	280.95 [248.27;320.28]	282.72 [247.30;322.58]	277.47 [237.48;310.91]	299.68 [257.34;335.11]	1.49E-05	0.000687
**Visceral fat**	14.44 [12.77;16.37]	14.56 [12.71;16.39]	13.84 [12.08;15.67]	15.61 [13.40;17.12]	1.01E-09	5.46E-08
Calcium	2.98 [2.94;3.02]	2.99 [2.96;3.02]	2.97 [2.94;3.00]	2.97 [2.94;3.00]	0.000229	0.008708
Phosphorus	3.40 [3.20;3.80]	3.45 [3.10;3.80]	3.40 [3.10;3.70]	3.40 [3.10;3.70]	0.13157	1
**Glucose**	4.55 [4.48;4.64]	4.54 [4.48;4.65]	4.54 [4.47;4.62]	4.58 [4.51;4.70]	0.000215	0.008374
BUN	2.71 [2.48;2.83]	2.70 [2.48;2.83]	2.71 [2.56;2.89]	2.71 [2.56;2.89]	0.004256	0.127674
**Uric acid**	2.51 [2.26;2.72]	2.54 [2.29;2.77]	2.40 [2.18;2.64]	2.52 [2.29;2.72]	1.68E-05	0.000757
Cholesterol	5.27 [5.12;5.41]	5.26 [5.12;5.41]	5.27 [5.12;5.39]	5.27 [5.14;5.39]	0.96966	1
Total protein	2.00 [1.96;2.03]	2.00 [1.96;2.03]	1.99 [1.95;2.03]	2.00 [1.96;2.03]	0.015994	0.430056
**Albumin**	6.12 [5.95;6.45]	6.29 [5.95;6.62]	6.12 [5.79;6.29]	6.12 [5.87;6.29]	1.20E-05	0.000576
Total bilirubin	0.47 [0.42;0.51]	0.45 [0.42;0.51]	0.47 [0.42;0.51]	0.45 [0.42;0.51]	0.94031	1
Alkaline phosphatase	4.11 [3.91;4.29]	4.14 [3.97;4.29]	4.06 [3.89;4.26]	4.13 [3.93;4.26]	0.006191	0.173345
AST	1.15 [1.14;1.16]	1.15 [1.14;1.17]	1.15 [1.14;1.16]	1.15 [1.14;1.17]	0.21967	1
ALT	3.09 [2.77;3.40]	3.14 [2.77;3.47]	3.00 [2.64;3.30]	3.04 [2.80;3.40]	0.00267	0.082782
**GGT**	3.14 [2.83;3.61]	3.22 [2.89;3.74]	3.00 [2.71;3.33]	3.26 [2.94;3.66]	1.55E-11	8.54E-10
TFT (free T4)	0.15 [0.08;0.22]	0.17 [0.06;0.24]	0.16 [0.08;0.23]	0.17 [0.09;0.25]	0.36042	1
TFTTSH	0.45 [0.19;0.83]	0.43 [0.09;0.78]	0.43 [0.18;0.71]	0.41 [0.19;0.73]	0.46153	1
OH-Vitamin D3	3.18 [2.92;3.38]	3.18 [3.00;3.35]	3.22 [2.98;3.41]	3.16 [2.92;3.34]	0.42693	1
Creatinine	-0.21 [-0.36;-0.06]	-0.21 [-0.37;-0.04]	-0.25 [-0.39;-0.09]	-0.16 [-0.30;-0.06]	0.005157	0.149565
Na	10224.00 [10081.50;10367.50]	10224.00 [10081.50;10367.50]	10224.00 [10081.50;10367.50]	10224.00 [10081.50;10367.50]	0.87829	1
K	4.10 [3.90;4.30]	4.10 [3.90;4.30]	4.10 [3.90;4.20]	4.10 [3.90;4.30]	0.53136	1
Cl	5617.50 [5407.50;5831.50]	5617.50 [5407.50;5831.50]	5617.50 [5407.50;5831.50]	5617.50 [5407.50;5831.50]	0.5861	1
Total CO2	315.56 [275.43;358.26]	315.56 [275.43;358.26]	315.56 [295.17;358.26]	315.56 [289.38;358.26]	0.66764	1
**Triglyceride**	95.00 [69.00;131.00]	102.00 [75.00;144.00]	84.00 [62.50;115.50]	97.50 [72.00;141.50]	1.15E-07	5.87E-06
HDL cholesterol	4.06 [3.89;4.25]	4.08 [3.89;4.23]	4.09 [3.91;4.28]	4.01 [3.85;4.20]	0.015928	0.430056
LDL cholesterol	4.74 [4.52;4.94]	4.72 [4.48;4.91]	4.75 [4.54;4.93]	4.73 [4.52;4.96]	0.55604	1
hsCRP	0.04 [0.01;0.09]	0.05 [0.01;0.11]	0.02 [0.01;0.08]	0.05 [0.01;0.10]	0.002355	0.077718
ESR	2.20 [1.77;2.55]	2.13 [1.77;2.59]	2.20 [1.77;2.59]	2.20 [1.88;2.51]	0.97956	1

**WBC**	1.67 [1.51;1.85]	1.72 [1.56;1.91]	1.65 [1.49;1.80]	1.71 [1.55;1.87]	6.02E-05	0.00247
**RBC**	4.70 [4.40;5.07]	4.74 [4.39;5.12]	4.58 [4.27;4.96]	4.79 [4.49;5.07]	1.29E-05	0.000607
**Hemoglobin**	26.14 [23.89;28.89]	26.14 [23.89;28.89]	25.23 [23.22;28.20]	26.82 [24.56;28.89]	3.81E-05	0.001638
**Hematocrit**	43.40 [40.70;46.70]	43.50 [40.70;46.90]	42.70 [39.65;45.80]	44.25 [41.70;46.75]	0.000134	0.005379
MCV	4333.30 [4112.74;4588.32]	4351.94 [4140.00;4588.32]	4398.72 [4185.62;4612.30]	4342.62 [4149.10;4550.08]	0.074023	1
RDW	0.50 [0.50;0.50]	0.50 [0.50;0.50]	0.50 [0.50;0.50]	0.50 [0.50;0.50]	0.058927	1
Platelet	5.46 [5.33;5.63]	5.49 [5.33;5.64]	5.46 [5.32;5.61]	5.46 [5.31;5.60]	0.44322	1
**HbA1c**	0.49 [0.49;0.49]	0.49 [0.49;0.49]	0.49 [0.49;0.49]	0.49 [0.49;0.49]	0.000676	0.024336
**Insulin**	2.02 [1.89;2.17]	2.04 [1.93;2.19]	1.97 [1.86;2.11]	2.07 [1.92;2.22]	9.93E-08	5.16E-06
AFP	0.89 [0.81;0.95]	0.89 [0.82;0.95]	0.89 [0.81;0.96]	0.90 [0.82;0.97]	0.62151	1
CEA	1.50 [0.90;2.10]	1.60 [1.00;2.30]	1.50 [0.90;2.20]	1.50 [1.00;2.30]	0.23074	1
PSA	1.39 [0.93;1.69]	1.33 [0.87;1.67]	1.48 [1.04;1.81]	1.35 [0.69;1.73]	0.002019	0.068643
CA 19-9	4.30 [1.90;10.70]	5.15 [2.30;12.30]	5.20 [2.20;13.95]	4.30 [1.80;8.90]	0.017391	0.434775
CA 125	2.54 [2.23;2.83]	2.62 [2.25;2.93]	2.46 [2.16;2.74]	2.51 [2.29;2.83]	0.002559	0.081888
BMI	3.16 [3.08;3.25]	3.16 [3.08;3.25]	3.15 [3.06;3.23]	3.20 [3.13;3.27]	1.55E-06	7.88E-05

Supplementary Table 3. Comparison of median values of clinical variables among the four Enterotypes.

Phenotype	Taxa	Beta	FDR_TMAT_F16
PC1	*Dorea_longicatena*	0.045004	0.000443
PC1	*Clostridium_glycyrrhizinilyticum*	0.038008	0.001873
PC1	*Ruminococcus_gnavus*	0.02934	0.019669
PC1	*Dorea_formicigenerans*	0.029287	0.022893
PC1	*Ruminococcus_faecis*	0.028765	0.024726
PC1	*Blautia_wexlerae*	0.026248	0.041047
PC1	*Holdemanella_biformis*	0.025713	0.041047
PC1	*Lactococcus_lactis*	-0.02701	0.029664
PC1	*Akkermansia_muciniphila*	-0.02787	0.024726
PC1	*Intestinibacter_bartlettii*	-0.03019	0.013833
PC1	*Ruminococcus_bromii*	-0.03296	0.012977
PC1	*Bacteroides_uniformis*	-0.03539	0.007136
PC1	*Ruminococcus_lactaris*	-0.03548	0.007778
PC1	*Anaerostipes_hadrus*	-0.0535	9.80E-06
PC4	*Ruminococcus_gnavus*	-0.08865	0.024152
PC4	*Eggerthella_lenta*	-0.10639	0.007052
PC5	*Blautia_faecis*	0.139343	0.003968
PC5	*Turicibacter_sanguinis*	0.101649	0.031565
PC5	*Eggerthella_lenta*	-0.09409	0.048332
PC5	*Clostridium_glycyrrhizinilyticum*	-0.1105	0.019669

Supplementary Table 4. Association test using TMAT between principal components (PCs) and 16S rRNA bacterial taxa.
